# Supplementary material for: Rare diseases in children—Knowledge, experiences and challenges faced by pediatricians in Tanzania
Source: PLOS Glob Public Health. 2026 May 13;6(5):e0006435. doi: 10.1371/journal.pgph.0006435 (PMC13170965; doi:10.1371/journal.pgph.0006435)
Supplement: S1 Text — (PDF) [file pgph.0006435.s002.pdf]

APPENDIX 1
QUESTIONNAIRE

Paediatricians Caring for Children with Rare Diseases: Survey of knowledge, practice, challenges, and future research.

Thank you for agreeing to complete this survey which will take 5-15 mins to complete. This survey is anonymous. Please click on the most appropriate answer to each question. For some questions you will be asked to comment - please write your comments in the box provided.

1. Gender:

☐ Male ☐ Female

2. Age:

☐ < 30 ☐30-35 ☐36-40 ☐41-45 ☐46-50 ☐51-55 ☐56-60 ☐61-64 ☐>65

3. In which year were you awarded your MMed (Pediatrics and Child Health) or equivalent?

____

4. In which City do you currently practice? _______________________

5. What best describes your practice ?

☐ Government based only ☐Private practice only ☐Combination of Government and Private practice

6. What category best describes your current practice?

☐ General Paediatrics

☐ Subspecialty Paediatrics

☐ Other

If others, please specify:_______________

7. How many new patients do you see in your practice in an average week

☐ < 1 / week ☐ 1-5 / week ☐5-10 /week ☐10-20 /week ☐>20 / week

8. What proportion of the general Tanzanian child population do you believe is affected by rare diseases?

☐ < 1% ☐ 2-3% ☐ 3-5% ☐ 6-8% ☐ 9-10% ☐ >10%

9. Have you ever looked after a child with a diagnosed/ undiagnosed rare disease or condition?

☐ Yes

☐ No

☐ May be

10. In your practice as a specialist/ consultant, please estimate how many ofyour patients have had a diagnosed rare disease/condition?

☐ 1-5 ☐ 5-10 ☐ 10-20 ☐ >20 ☐ >100

11. What types of rare conditions have you been involved in managing? (tick as many as apply)

☐ Metabolic/Genetic
☐ Renal
☐ Gastrointestinal
☐ Endocrine
☐ Neurological
☐ Connective tissue
Rheumatic
☐ Musculoskeletal
☐ Respiratory
☐ Infectious
☐ Immunological
☐ Dermatological
☐ Cardiac
☐ Cancer
☐ Other
If Other, Please specify ________________________

12. In your practice have you ever looked after a patient with an unusual cluster of signs and symptoms for whom a definitive diagnosis was hard to establish?

☐ Yes ☐ No ☐ May be

13. Can you estimate how many such patients who have no definitive diagnosis you have managed throughout your clinical career as a Specialist/ Consultant?

☐ 1-5 ☐ 5-10 ☐10-20 ☐ >20

14. When did you last see a new patient with either a diagnosed rare disease or an undiagnosed cluster of signs and symptoms?

☐ < 6 months ago ☐ 6-12 months ago ☐ 1-3 years ago ☐ >3 years ago

15. When diagnosing and/or managing patients with rare diseases or conditions, in which of the following areas have you encountered difficulties? (select as many as apply)

☐ Lack of access to diagnostic/genetic tests

☐ Delay in, or inability to make a definitive diagnosis

☐ Lack of management guidelines

☐ Lack of available treatments

☐ Uncertainty about where to refer to

☐ Difficulties in accessing specialised clinics/services

☐ Difficulties in accessing allied health services (e.g. physio, speech, OT, psychology etc.)

☐ Uncertainty about available peer support groups for the patient and his/her family

☐ Other difficulties

If others, please specify___________________

16. Were rare childhood diseases covered in your Medical Degree (undergraduate) curriculum?

☐ Yes ☐No ☐ May be

17. Did your Residency training adequately cover rare childhood diseases, to enable you to effectively provide patient care?

☐ Yes ☐ No ☐ May be

18. Have you attended any educational lectures/workshops about rare diseases in the last 5 years?

☐ Yes ☐No ☐ May be

If yes were these workshops/conferences useful?

☐ Yes ☐ No ☐ May be

What you find most useful? (tick all that apply)

☐ Content information
☐ Networking opportunities
☐ Resources
☐ Other Please specify:________________

19. In your practice, do you have reliable access to the internet?

☐ Yes ☐ No

20. Have you used internet resources to guide your patient management?

☐ Yes ☐ No

21. In your everyday Paediatric practice do you use a smartphone (e.g. iPhone or Android) or tablet/iPad?

☐ Yes ☐ No

22. Please indicate which of the following resources you have heard of, used and found helpful in your paediatric practice? (tick across all applicable columns).

☐ PubMed, Medline, or similar

☐ BMJ Best Practice guidelines

☐ CDC National GuidelinesClearing House

☐ National Institutes for Health andClinical Excellence (NICE; UK)

☐ ORPHANET portal for rarediseases

☐ Online Mendelian Inheritance inMan (OMIM)

☐ NORD (National Organisation forRare Diseases)

☐ Up-To-Date

☐ Cochrane Library

☐ European Organisation for RareDiseases (EURORDIS)

☐ POSSUM (Pictures Of StandardSyndromes and Undiagnosed Malformations)

☐ Others

If Others, please specify_________________________

23. Which of the following resources do you routinely use in your everyday practice to help you diagnose and manage your paediatric patients? (select all applicable)

☐ Textbooks

☐ Consultation with colleagues

☐ Referral to specialist clinics/tertiary centres

☐ Web-based resources

☐ Apps on smartphones or other mobile devices (e.g. IPhone; Blackberry; tablet/iPad)

☐ Other

If Other, please specify _______________________________

24. What types of educational resources about rare diseases are you likely to use if these were available?

☐ Online modules via the MCT website about how to use already existing on-line resources

☐ Face-to-face educational workshops/seminars about howto use already existing on-line resources

☐ Smart phone/tablet applications about how to use already existing on-line resources

☐ Online modules via the MCT website about specific rare diseases or groups of rare diseases

☐ Face to face educational workshops/seminars about specific rare diseases or groupsof rare diseases

☐ Printed materials/modules about specific rare diseases or groups of rare diseases

☐ Smart phone/tablet applications on rare disease diagnosis and management

☐ A web portal providing a listing of specialists and specialist clinics you can refer your patients to

☐ A web portal providing a listing of support groups available for your patients and their families

☐ A web portal providing fact sheets about specific rare diseases which you could give to your patients

25. What would make you more likely to regularly access already existing web-based resources on

rare diseases in your clinical practice?

- ☐ Know-how on what each web-based resource offers, how to access and how to use them, to optimise your clinical practice
- ☐ A single web portal listing all available web-based resources with links to these resources
- ☐ Fast point-of-care (mobile) access to each site (eg. iPhoneapplication)
- ☐ Up-to-date, clinically reliable content
- ☐ Free access/affordable subscription
- ☐ Regular updates on web-based resources delivered to your email

26. To what extent do you agree with the following statements:

	Strongly agree	Agree	Disagree	Strongly disagree
I feel that my medical degree training was adequate for me to recognise patients with rare diseases				
I don't think I need training about how to use databases and other on-line resources about rare diseases				
I feel confident that I would know the referral pathway whenfaced with a rare disease patient				
I feel confident that I would know where to find informationabout diagnosis and management of rare diseases				
Rare diseases are not important				
I believe that patients with rare diseases are best looked after in				

a multidisciplinary clinic

I feel unprepared to look after patients with rare diseases

I have adequate access to experts in rare diseases who advise me about management of rare disease patients

Thank you for taking part in this survey
