# Supplementary material for: Rare diseases in children—Knowledge, experiences and challenges faced by pediatricians in Tanzania
Source: PLOS Glob Public Health. 2026 May 13;6(5):e0006435. doi: 10.1371/journal.pgph.0006435 (PMC13170965; doi:10.1371/journal.pgph.0006435)
Supplement: Attachment — Submitted filename: PGPH-D-25-00096_R2_reviewer.pdf [file pgph.0006435.s006.pdf]

PLOS Global Public Health

Rare diseases in children - knowledge, experiences and challenges faced by Pediatricians in Tanzania

--Manuscript Draft--

**Manuscript Number:**	PGPH-D-25-00096R2
**Article Type:**	Research Article
**Full Title:**	Rare diseases in children - knowledge, experiences and challenges faced by Pediatricians in Tanzania
**Short Title:**	Rare diseases in Tanzania
**Corresponding Author:**	Mariam Noorani, MMed<br>Aga Khan University - Tanzania<br>Dar es Salaam, TANZANIA, UNITED REPUBLIC OF
**Order of Authors:**	Mariam Noorani, MMed
	Mohamedraza Ebrahim
	Francis Furia
**Keywords:**	rare diseases; Children; pediatricians; Tanzania; challenges; Knowledge; Experiences
**Abstract:**	Diagnosing and treating rare diseases in children is a major challenge for pediatricians across the world. There is a lack of adequate knowledge of these conditions and diagnostic testing is not easily accessible, which frequently results in delays in care. The knowledge, experiences and challenges faced by pediatricians in Tanzania is not known. This study presents the results of a nationwide cross-sectional survey which aimed to describe the knowledge of pediatricians in Tanzania on rare diseases, their experiences, and the challenges they face in treating these children. Of the 323 pediatricians registered on the Pediatric Association of Tanzania WhatsApp group, which is about 90% of pediatricians registered to practice in Tanzania, 168 completed the survey, giving a response rate of 52%. All of them had encountered a child with rare diseases in their career, with 60% having seen one in the 6 months preceding the survey. The commonest rare condition encountered was genetic/metabolic, and the most common difficulty (97%) encountered was lack of access to diagnostic testing. Only a third of respondents reported that rare diseases were taught in university and 60% of felt unprepared to look after these children. Three quarter of respondents did not have access to experts to advise them on management. Rare diseases are commonly encountered by pediatricians in Tanzania, and there is a challenge in diagnostic testing as well as a gap in training, with pediatricians not confident in providing care. They also lack access to experts who can advise on rare disease management. There is an urgent need to find ways to make diagnostic testing available, accessible and affordable. A review of medical training curricula should be done to incorporate rare disease education and skill development. Strategies to connect pediatricians with regional and global experts should be put in place. The study findings may be limited by the low response rate, the use of technology to distribute the survey and imbalance of sub-specialty and general practice.
**Additional Information:**	
**Question**	**Response**
**Financial Disclosure**<br><br>Enter a financial disclosure statement that describes the sources of funding for the work included in this submission and the role the funder(s) played. This includes	Partial funding for this work was provided by the Ali Kimara Rare Disease Foundation. The funders had no role in study design, data collection and analysis, decision to publish, or preparation of the manuscript. No additional external funding was provided for this study

Powered by Editorial Manager® and ProduXion Manager® from Aries Systems Corporation

grants and any commercial funding of the work or authors.<br><br>This statement will be typeset if the manuscript is accepted for publication.<br><br>*Please review the submission guidelines and the instructions link below for detailed requirements and guidance.*	
**Competing Interests**<br><br>On behalf of all authors, disclose any competing interests that could be perceived to bias this work.<br><br>This statement will be typeset if the manuscript is accepted for publication.<br><br>*Please review the instructions link below and PLOS Global Public Health's competing interests policy to determine what information must be disclosed at submission.*	Two of the authors: MN and MA are board members of the Ali Kimara Rare Disease Foundation which provided partial funding for the study. These interests do not alter the authors' adherence to PLOS Global Public Health policies on sharing data and materials.
**Data Availability**<br><br>Before publication, Authors are required to make fully available and without restriction all data underlying their findings. Please see our PLOS Data Policy page for detailed information on this policy.<br><br>A **Data Availability Statement**, detailing where the data can be accessed, is required at first submission. Insert your Data Availability Statement in the box below.<br><br>Please see the data reporting section of our submission guidelines for instructions on what you need to include in your Data Availability Statement.	The dataset used for this study has been provided as supplementary material.

This statement will be typeset if the manuscript is accepted for publication.<br><br>*PLOS allows rare exemptions to address legal and ethical concerns. If you have legal or ethical restrictions, please use the box below to detail these in full sentences for the Journal team to consider.*	

Powered by Editorial Manager® and ProduXion Manager® from Aries Systems Corporation

Manuscript Click here to access/download;Manuscript;manuscript.docx

Rare diseases in children - knowledge, experiences and challenges faced by Pediatricians in Tanzania

Mariam Noorani[1,2*], Mohamedraza Ebrahim [1], Francis Furia [2]

[1]Aga Khan University, Department of Pediatrics, Dar es Salaam, Tanzania

[2]Muhimbili University of Health and Allied Sciences, Department of Pediatrics and Child Health, Dar es Salaam, Tanzania.

*** Corresponding author**

email: mariam.noorani@aku.edu (MN)

All authors contributed equally to this work

Keywords: rare diseases, children, pediatricians, Tanzania, challenges, knowledge, experiences.

Abstract

Diagnosing and treating rare diseases in children is a major challenge for pediatricians across the world. There is a lack of adequate knowledge of these conditions and diagnostic testing is not easily accessible, which frequently results in delays in care. The knowledge, experiences and challenges faced by pediatricians in Tanzania is not known. This study presents the results of a nationwide cross-sectional survey which aimed to describe the knowledge of pediatricians in Tanzania on rare diseases, their experiences, and the challenges they face in treating these children. Of the 323 pediatricians registered on the Pediatric Association of Tanzania WhatsApp group, which is about 90% of pediatricians registered to practice in Tanzania, 168 completed the survey, giving a response rate of 52%. All of them had encountered a child with rare diseases in their career, with 60% having seen one in the 6 months preceding the survey. The commonest rare condition encountered was genetic/metabolic, and the most common difficulty (97%) encountered was lack of access to diagnostic testing. Only a third

of respondents reported that rare diseases were taught in university and 60% of felt unprepared to look after these children. Three quarter of respondents did not have access to experts to advise them on management. Rare diseases are commonly encountered by pediatricians in Tanzania, and there is a challenge in diagnostic testing as well as a gap in training, with pediatricians not confident in providing care. They also lack access to experts who can advise on rare disease management. There is an urgent need to find ways to make diagnostic testing available, accessible and affordable. A review of medical training curricula should be done to incorporate rare disease education and skill development. Strategies to connect pediatricians with regional and global experts should be put in place. The study findings may be limited by the low response rate, the use of technology to distribute the survey and imbalance of sub-specialty and general practice.

Introduction

Rare diseases are a group of medical conditions that affect a small number of people in the population. Definitions of rare diseases vary globally, and different criteria are used to describe these conditions. In the United States, a condition affecting less than 200,000 people is considered rare, while in Europe rare disease is one affecting less than 5 in 10,000 people. [1]

Globally, there are between 5000 to 8000 rare diseases that have been identified affecting between 350 and 475 million people. The burden of these conditions is presumed to be higher in African countries, which have limited capacity to diagnose these patients, as well as a lack of treatment options and high cost of care, resulting in economic hardships, severe complications, and subsequent disabilities and mortality. [2]

The diagnosis of these conditions depends largely on laboratory and genetic testing because of limited clinical experience with their manifestations, resulting in delayed diagnosis and treatment. Lack of

treatment guidelines and the rarity of expert clinicians with experience managing these conditions further compounds the problem. [3].

In developed countries, Paediatric sub-specialists, including geneticists, hematologists and endocrinologists, provide care to children with rare diseases. However, in low and middle income settings, primary care providers, including medical officers and pediatricians are on the front line of care for affected children [4]. Medical officers and pediatricians in low resource settings communicate with parents regarding diagnosis and potential management and coordinate referral to tertiary facilities for advanced management. Therefore, they need to have adequate knowledge to provide counselling, diagnostic information and care for families, particularly for conditions whose therapy is not accessible or not yet developed.

However, global data suggest that pediatricians and other specialist doctors are not adequately trained to recognize, diagnose, or manage these conditions. A nationwide survey of pediatricians from Australia showed that only about 50% of them reported that rare diseases were adequately covered during their training, while about a quarter felt unprepared to care for these children. [5] Similarly, over 90% of physicians undertaking specialization in Poland perceived their knowledge on rare diseases as poor and less than 5% felt confident of taking care of these patients. [6] A study from Spain found that less than a third of physicians reported receiving training on rare diseases during their undergraduate and postgraduate years. [7]

Very few studies gauging healthcare provider knowledge are available from the developing world. One survey from India reported that only a third of physicians surveyed had some knowledge of rare diseases while almost 80% reported they had no resources to diagnose and manage these conditions and their institution had no continuing education program in rare diseases. [8]

Apart from lack of adequate knowledge, there are many challenges that pediatricians face in the diagnosis and management of rare diseases. Studies from high-income countries, including Australia, have identified diagnostic delays, lack of available treatments, and uncertainty of where to refer as common challenges. [5] Lack of specialists was highlighted as a challenge in in Malaysia by Shafie et al, reporting only 13 rare disease specialists in the country. This study also showed that about 40% of patients with rare diseases were on symptomatic treatment only. [9]

Various interventions have been proposed to improve access to knowledge and improve the diagnosis of rare diseases. Physicians in a Belgian study recommended specific education on "red flags" or pointers to increase attentiveness to rare diseases. [10] Use of specific platforms like Orphanet and other rare disease online resources is another potential way to improve knowledge and its application towards timely identification of rare diseases.

In recent years, patient advocacy groups have accelerated the global progress in diagnosing and managing rare conditions by bringing together governments, patients and scientific communities. [3] The coming together of researchers and clinicians to form consortia has played a major role in furthering research and treatment of rare diseases, and pediatricians should be an integral part of such consortia.

In Tanzania, information is scarce on rare diseases in children; the epidemiology, the types and the management options are not known. Since 2016, the country has been commemorating the World Rare Disease Day, which has been driven by a patient advocacy group called Ali Kimara Rare Disease Foundation (AKRDF). This group brings together families affected by rare diseases and well-wishers from different sectors including media and sports who raise awareness on the plight of families. The rare disease agenda in the country has further been driven by the formation of the Tanzania Human Genetics Organization (THGO) in 2019 which aims to bring together various stakeholders in the field

of genetics. [11] These include geneticists, lab scientists, pharma experts and doctors who provide technical expertise, undertake research and advise communities and government on policy related to rare diseases.

In 2020, a national call to action was made during the commemoration of World Rare Disease Day that was co- hosted by the THGO and AKRDF. The call highlighted various areas of urgent action including identifying demographics of patients with rare diseases, developing guidelines for diagnostics and treatment as well as strengthening research. [12]

The rare occurrence of these diseases, the lack of epidemiologic data and unavailability of diagnostic testing and treatment guidelines may limit pediatricians in their ability to identify these patients. Many of these conditions, especially in the perinatal period present with signs and symptoms that may mimic other conditions. [13]

In Tanzania, there is no data on how frequently pediatricians encounter rare diseases in their practice, their knowledge of these conditions and the challenges that they face in management.

This study aimed to determine the knowledge, experiences and challenges perceived by pediatricians in Tanzania in managing presumed rare diseases. This is the first step in highlighting the burden of the problem. The data from this study will help to identify specific needs for training to improve knowledge and skills of pediatricians in managing children with rare diseases. Additionally, the challenges cited will prioritize areas for improvement. The data can also be used to inform stakeholders in medical education to include conditions from this group in the curriculum for medical training.

Materials and Methods

Study design, setting and population: This was a nationwide cross-sectional survey conducted among pediatricians working in Tanzania (Mainland and Zanzibar) who are members of Paediatric Association of Tanzania (PAT) and were included in the official PAT WhatsApp social media platform. PAT is the official professional association operating in United Republic of Tanzania, it had 325 members at the time of conducting this study (informal communication with PAT leadership), who were licensed and practicing at all levels of the health-care system from primary health care to tertiary referral facilities. The PAT WhatsApp platform is utilized for communication among members for social, academic and clinical issues. Members of the group are strictly pediatricians and are added soon after graduation. Over 90% of the pediatricians in the country are part of the group; those who are not are either non-practicing or choose to stay away from social media. The platform is also used for seeking consultation and discussion of cases managed by pediatricians who are in the group, discussions about patients are conducted with strict adherence to ethical principles of not sharing information that could reveal patients' identities. We used the WhatsApp group to disseminate this survey since it provided the widest reach to the target population.

Data collection instruments: Data was collected using a questionnaire (attached as supporting information) which was adapted from the instrument used in a similar study conducted in Australia. [5] This tool was found to be appropriate for this study because it provided comprehensive questions that aligned with the objectives of our research, making it a suitable foundation for adaptation. Questions were modified to fit the Tanzanian healthcare context; modifications mainly involved changes in geographical regions, regulatory authorities and access to relevant health care information. The modified questionnaire included sections on:

1. Pediatricians' demographics and practice which asked questions about years of experience, site of practice and sub-specialty training

2. Pediatricians’ experience with rare disease patients including types of rare diseases, how many patients they had seen and how frequently they manage them
3. Challenges encountered by pediatricians in managing children with rare diseases.
4. Pediatricians’ education, training and their self-reported knowledge of rare diseases which asked about rare disease education during undergraduate and postgraduate training as well as workshops or seminars related to rare diseases
5. Likelihood of use of resources which included questions about their preferred resource with information about rare diseases
6. Pediatricians’ perceptions and confidence regarding providing care to children with rare diseases which asked about access to experts, knowing the pathway to channel a child with rare disease and their preparedness to provide care

Data collection process: The questionnaire was pilot-tested on 4 senior paediatric residents (postgraduate trainees) to ensure the meaning, content and flow of the questions was clear. We selected senior paediatric residents because they were almost graduating and would have relevant experience in understanding concepts and questions related to rare diseases. The online link was shared with them, they reviewed it, filled out all the sections and gave written feedback on each section of the questionnaire. Adjustments that were made based on their input included the flow and structure of the questions and additions to the list of available resources.

The questionnaire was then shared as an online link circulated to members in the WhatsApp platform. To encourage participation in the survey, zonal PAT representatives were informed about the survey and requested to remind their members to accept the link and respond to the survey. All pediatricians on the group were eligible to take the survey as long as they were in active clinical practice, this was indicated at the beginning of the online questionnaire.

Data handling and analysis: Data was collected between 7th January 2024 and 4th February 2024 and was auto-populated onto a Microsoft online Excel sheet. The data were checked for completeness and consistency and subsequently coded and analyzed using inbuilt formulae in Excel. Missing data in specific sections was noted and was omitted during analysis. Data were summarized into frequency distribution tables and charts that described the responses of participants regarding various aspects of rare diseases.

Ethical considerations: Ethical clearance was obtained from the Muhimbili University of Health and Allied Sciences (MUHAS) Institutional Review Board (ref: DA. 282/298/01.C/1895) and the permission to approach pediatricians was obtained from the professional society: Pediatric Association of Tanzania (PAT). The introductory section of the questionnaire provided information related to confidentiality, risks and benefits (Image provided as supporting information). Written consent was not obtained but was implied if the pediatrician filled out the online form and submitted it after reviewing the information provided.

Confidentiality was maintained by not collecting data on any direct identifiers like names or email addresses. Study data was kept confidential and accessible only to the investigating team by use of password protected computers.

Results

A total of 168 pediatricians completed the survey giving a response rate of about 52%. There was representation from all zones in the country with the majority (63%) coming from the Eastern zone where the commercial capital city – Dar es Salaam is located. Figure 1 shows the distribution of respondents from different zones of the country which is in keeping with the distribution of pediatricians in these areas

Fig 1. Distribution of respondents from different zones in Tanzania.

The map illustrates percentage of respondents from the different geographical zones in Tanzania. Map modified from Mzee, 2021 [14] by adding percentages. Available from: https://doi.org/10.1186/s42269-021-00612-z. Available via cc by 4.0 licence: http://creativecommons.org/licenses/by/4.0/.

Most participants (77%) were women and 79% were under the age of 45 years. Only about 30% had more than 10 years of clinical experience in the field of pediatrics. About half of the respondents worked in both public and private health care settings and the majority (81%) were general pediatricians with only a fifth being sub-specialized in a specific field of pediatrics. The characteristics of the participants are summarized in Table 1.

Table 1: Characteristics of study participants

VARIABLE	N (%)
Gender	
Male	39 (23%)
Female	129 (77%)
Age groups	
<= 45 years	132 (79%)
46-60 years	31 (18%)
>60 years	5 (3%)
Years of experience as a pediatrician	
<5 years	60 (36%)
5 - 10 years	52 (31%)
>10 years	53 (31%)
Missing data	3 (2%)
Type of practice	
Public sector only	30 (18%)
Private sector only	44 (26%)

Both public and private sector	94 (56%)
Specialty	
General pediatrics	136 (81%)
Pediatric Sub-specialty	32 (19%)

When asked how frequently rare diseases occur in Tanzania, most of the respondents (81%) believed that less than 5% of the Tanzanian child population is affected by rare diseases. Regarding their experiences in seeing children with rare diseases, all respondents had encountered a child with rare disease during their career but only 25 (15%) had seen more than 20 such children. Of these, about half of them were sub-specialists. Almost all (94%) reported looking after a child with an unusual cluster of symptoms and signs whose diagnosis could not be established and 22% reported having seen more than 10 such children. The most frequently encountered rare conditions were metabolic/genetic (74%) followed by neurological, musculoskeletal and cardiac. Table 2 summarizes the experiences in encountering rare diseases.

Table 2: Experiences of pediatricians in encountering rare diseases

**VARIABLE**	**N (%)**
Number of patients looked after with diagnosed rare diseases throughout career	
1-5 patients	88 (52%)
6-10 patients	38 (23%)
11-20 patients	17 (10%)
>20 patients	25 (15%)
The number of patients looked after without definitive diagnosis reached	
1-5 patients	94 (56%)
6-10 patients	36 (22%)
11-20 patients	19 (11%)
>20 patients	19 (11%)

Types of rare conditions encountered[1]	
Metabolic/genetic	124 (74%)
Neurological	90 (54%)
Musculoskeletal	59 (35%)
Cardiac	59 (35%)
Endocrine	55 (33%)
Immunological	53 (32%)
Dermatological	54 (32%)
Gastrointestinal	47 (28%)
Respiratory	46 (27%)
Cancer	45 (27%)
Connective tissue	42 (25%)
Renal	38 (23%)
Rheumatic	33 (20%)
Infectious	26 (16%)
Hematologic	2 (1%)
When the most recent suspected or diagnosed rare condition was seen	
< 6 months ago	101 (60%)
6-12 months ago	46 (27%)
1-3 years ago	12 (7%)
>3 years ago	9 (6%)

[1] Multiple selection was allowed for this question

When asked about challenges encountered while managing children with rare diseases, the commonest difficulty was lack of access to diagnostic and genetic tests with 97% of pediatricians encountering this difficulty. Other common challenges encountered included a lack of treatments (70%) and delay or inability to make a definitive diagnosis (66%). The challenges encountered are summarized in figure 2.

Figure 2. Challenges encountered by pediatricians in managing rare diseases

Regarding training received on rare diseases, only about a third of respondents said rare diseases were taught during their undergraduate curriculum and a similar proportion reported being taught during their postgraduate training. Only 30% of them had ever attended any workshops or lectures related to rare diseases.

On exploring their perceptions and confidence in recognizing and managing rare diseases, only about half of the respondents felt that their training was adequate for them to recognize patients with rare diseases and about 65% felt confident that they would know the referral pathway for a child with rare disease. About two-thirds (60%) felt unprepared to look after patients with rare diseases and over 70% did not have access to experts to support them in providing care. Responses to other sections of the questionnaire including confidence in finding rare disease information and perception on role of multidisciplinary care are further described in Figure 3.

Figure 3. Perception and confidence in caring for children with rare diseases

When asked about the likelihood of using resources with information on rare diseases, over 80% of respondents favored a smartphone or tablet application while about 60% would use online modules via the Medical Council of Tanzania (MCT) website and a similar percentage would utilize face-to-face workshops or seminars. The likelihood of resource use by respondents are summarized in figure 4.

Figure 4. Likelihood of resource utilization of pediatricians managing rare diseases

Discussion

This study was conducted to determine knowledge, experiences and challenges in managing rare diseases among pediatricians in Tanzania. To the best of our knowledge, this is the first research study to investigate the experience of health care providers in managing rare diseases in Tanzania, the challenges faced in their treatment and the education and training about these conditions. There have been case reports and case series from Tanzania on individual rare diseases or rare diseases related to

specific systems like rheumatology [15-17], however the overall spectrum of rare diseases has not been described. The findings of our study, having focused on pediatricians who are the specialists most likely to encounter rare conditions, can act as a proxy of the situation of rare disease knowledge, experiences and challenges in Tanzania.

All participants in our study reported having managed rare disease patients during their career; however, only 25% of participants had managed more than 10 children with rare diseases. These findings reflect on the overall prevalence of these conditions; individually they may be rare, but as a group they are encountered in clinical practice although infrequently; similar to the findings by Rohani-Montez et al in a study conducted among clinicians in Europe and the United States where more than half reported that they have rarely (1-2 patients per year) seen patients with a rare disease. [18]

It is plausible to attribute the reported experience of managing a higher number of children to sub-specialty training; 19% of participants had sub-specialty training while 25% of participants had managed more than 10 children with rare disease. Healthcare providers with sub-specialty and /or multiple specialties have better knowledge and experience in managing children with rare diseases and are usually practicing in referral and specialized healthcare facilities. [19]

Limited access to diagnostic testing was the challenge most commonly faced by the participants in this study. This is because of lack of availability of genetic testing in Tanzania and the added high cost of testing if the sample is sent outside the country. The difficulties in accessing genetic testing has been highlighted as a problem even in high income countries as reported by Zurynski in Australia. [5] Additionally, pediatricians in Australia also reported delays in interpretation of genetic tests; highlighting that apart from the physical diagnostic infrastructure, equally important is the human resource of clinical geneticists. In Tanzania, clinical genetics is a specialization that is not readily available with only 1 specialist in the country.

Comparative data from other low- and middle-income countries (LMICs) highlight similar challenges in the diagnosis and management of rare diseases in children. Studies conducted in LMICs indicate that pediatricians in these regions face significant barriers, including limited access to diagnostic tools, a lack of specialized expertise, and inadequate training on rare diseases—findings that closely align with our study in Tanzania. For instance, research from sub-Saharan Africa and Southeast Asia has noted the scarcity of genomic sequencing availability and the higher costs associated with diagnostic testing, contributing to delayed diagnosis and treatment. [20]

Pediatricians in this study expressed a lack of training on rare diseases in both undergraduate and postgraduate training. There was also poor confidence in diagnosing and managing children with rare diseases with the majority indicating a lack of confidence. Similar findings have been documented from other studies globally, indicating low knowledge and ability to diagnose patients; this has consequently resulted in delays in diagnosis and treatment. [21-22] There is also lack of awareness of the global epidemiology with most pediatricians believing that less than 5% of Tanzanian children are affected by rare diseases; which is lower than the estimated global prevalence of rare diseases of between 6-8%.

Capacity building for managing children with rare diseases in the form of guidelines, treatment, and care pathways with improvement in diagnostic facilities including treatment networks have been advocated for in several studies. [23, 24] Participants in our study suggested various methods that can be used to improve their knowledge and skills on rare diseases. Use of a smart phone application with all relevant information on rare diseases is the resource most favored by pediatricians in Tanzania. This is likely due to the current age of digitalization and familiarity with technology. Majority of our study participants were less than 45 years of age and this is a generation which is comfortable and confident in using smart phone applications.

Other resource options include physical and virtual workshops, and availability of materials through web portals. Similar approaches were reported in an online survey which was conducted among clinicians in Europe and the United States who preferred a comprehensive online learning platform with current education on rare diseases. [17] It is therefore imperative for stakeholders involved in the care of these children including patient support groups, paediatric associations, medical schools, hospitals and the Ministry of Health to strategize and organize coordinated educational programs.

Most participants in this study agreed that children with rare disease should be managed by a multidisciplinary team, however less than a quarter of them had access to experts who could advise on the management of these patients. This shows a possible gap in coordinated care at different levels of the health system but could also be a reflection of lack of specialized pediatricians with the required knowledge and skills to assess and manage rare diseases. Globally, there has been a move to develop rare disease clinical networks that span across continents and harness the expertise of clinicians and researchers to provide the best possible care to patients. [25]

Rare diseases cause significant burden to the patients, families, communities and health systems. Diagnostic delay is costly to the families and health systems and this is worsened by the poor knowledge and skills of practitioners. [18, 26, 27] These diseases contribute significantly to morbidity and mortality among children including infants and neonates, [28] it is therefore important to consider strategies for mitigating existing challenges with a priority on capacity building for healthcare providers.

Our study has some limitations to note. Firstly, the response rate was 52% hence may not be reflective of all pediatricians in Tanzania. Secondly, given that a significant proportion (79%) of respondents were under 45 years of age, there is a possibility that the study may be biased toward younger pediatricians. Thirdly, among the respondents, pediatricians with sub-specialty training were 19% and they are more likely to recognize and manage rare disease compare to general pediatricians hence this

may bias the findings. Finally, the use of an online survey via the PAT WhatsApp group may have excluded some eligible participants who were not on the group, as well as those who were not familiar or comfortable with the use of technology to complete online surveys.

To address these limitations, future research could aim for a more representative sample, incorporating strategies to enhance participation from older pediatricians. Additionally, further studies could explore whether digital tools are equally acceptable across different age groups. Despite these limitations, the study provides valuable insights into the challenges faced by pediatricians regarding rare diseases and highlights a significant gap in training and diagnostic resources.

Conclusions and Recommendations

Our findings suggest that pediatricians in Tanzania frequently encounter children with presumed rare diseases, with genetic/metabolic conditions being the most common from their perspective. However, a significant challenge lies in the limited access to diagnostic testing and lack of experts, which hinders effective management of these conditions. Furthermore, there is a notable gap in the training on rare diseases, leaving many pediatricians feeling unprepared to care for these children.

To improve the care of children with rare diseases in Tanzania, there is urgent need to improve access to diagnostic testing and reduce the cost by providing subsidies and creating mechanisms through which these tests can be easily requested when needed.

A review of undergraduate and postgraduate medical curricula should be done to incorporate rare diseases as an important component of training. Access to relevant expertise and resources should be prioritized and networks of care developed to connect pediatricians with national, regional and global experts.

We recommend that future studies should focus on developing a registry to document the true spectrum of rare diseases in Tanzania. Population based studies should be conducted to quantify the prevalence

and burden of rare diseases and genetic and genomic research should be encouraged for accurate identification and risk stratification. Exploring the experiences of families of children with rare diseases is equally important to identify their challenges and needs for better support systems.

Acknowledgments

The authors acknowledge the support from the Paediatric Association of Tanzania in conducting this study.

References

1. Richter, T., Nestler-Parr, S., Babela, R., Khan, Z. M., Tesoro, T., Molsen, E., & Hughes, D. A. (2015). Rare Disease Terminology and Definitions—A Systematic Global Review: Report of the ISPOR Rare Disease Special Interest Group. Value in Health, 18(6), 906–914. https://doi.org/10.1016/j.jval.2015.05.008

2. Adachi T, El-Hattab AW, Jain R, Nogales Crespo KA, Quirland Lazo CI, Scarpa M, Summar M, Wattanasirichaigoon D. Enhancing equitable access to rare disease diagnosis and treatment around the world: a review of evidence, policies, and challenges. Int J Environ Res Public Health. 2023 Mar 8; 20(6):4732.

3. Stoller J. K. The Challenge of Rare Diseases. CHEST. 2018; 153(6), 1309–1314.

4. Baynam, G., Hartman, A. L., Letinturier, M. C. v, Bolz-Johnson, M., Carrion, P., Grady, A. C., Dong, X., Dooms, M., Dreyer, L., Graessner, H., Granados, A., Groza, T., Houwink, E., Jamuar, S. S., Vasquez-Loarte, T., Tumiene, B., Wiafe, S. A., Bjornson-Pennell, H., & Groft, S. (2024). Global health for rare diseases through primary care. The Lancet Global Health, 12(7), e1192–e1199. https://doi.org/10.1016/S2214-109X(24)00134-7

5. Zurynski Y, Gonzalez A, Deverell M, Phu A, Leonard H, Christodoulou J, & Elliott E. Rare disease: a national survey of pediatricians' experiences and needs. BMJ Paediatr Open. 2017; 1(1). https://doi.org/10.1136/BMJPO-2017-000172

6. Walkowiak D, & Domaradzki J. Are rare diseases overlooked by medical education? Awareness of rare diseases among physicians in Poland: an explanatory study. Orphanet J Rare Dis. 2021; 16(1), 1–12.

7. Ramalle-Gómara E., Domínguez-Garrido E, Gómez-Eguílaz M, Marzo-Sola M. E, Ramón-Trapero J. L & Gil-De-Gómez J. Education and information needs for physicians about rare diseases in Spain. Orphanet J Rare Dis. 2020; 15(1), 1–7.

8. Agrawal R, Amaresh Rao M, Brian M, Chowdary G, Gayatri K, Krishnaji Rao M. Baseline knowledge of rare diseases in India—a survey. Int J Rare Dis Disord. 2019; 2(1):4-11.

9. Shafie A. A, Supian A., Ahmad Hassali M. A, Ngu L. H, Thong M. K, Ayob H, & Chaiyakunapruk N. Rare disease in Malaysia: Challenges and solutions. PloS one. 2020; 15(4), e0230850. https://doi.org/10.1371/journal.pone.0230850

10. Vandeborne L, van Overbeeke E, Dooms M, de Beleyr B, & Huys I. Information needs of physicians regarding the diagnosis of rare diseases: A questionnaire-based study in Belgium. Orphanet J Rare Dis. 2019; 14(1), 1–11.

11. Alimohamed MZ, Mwakilili AD, Mbwanji K, Manji ZK, Kaywanga F, Mwaikono KS, Adolf I, Makani J, Hamel B, Masimirembwa C, Ishengoma DS. Inauguration of the Tanzania Society of Human Genetics: biomedical research in Tanzania with emphasis on human genetics and genomics. Am J Trop Med Hyg. 2021; Feb; 104(2):474.

12. Kaywanga F, Alimohamed MZ, David AB, Maeda D, Mbarak S, Mavura T, Nkya S, Ishengoma DS. Rare diseases in Tanzania: a National Call for Action to address policy and urgent needs of individuals with rare diseases. Orphanet J Rare Dis.2022. Sep 5; 17(1):343.

13. Bordini, B. J., & Kliegman, R. M. (2020). Undiagnosed and Rare Diseases in Perinatal Medicine: Uncommon Manifestations or Mimics of Neonatal Disorders. *Clinics in Perinatology*, *47*(1), xxi–xxii. https://doi.org/10.1016/j.clp.2019.12.001

14. Mzee, T., Kazimoto, T., Madata, J. et al. Prevalence, antimicrobial susceptibility and genotypic characteristics of Staphylococcus aureus in Tanzania: a systematic review. Bull Natl Res Cent 45, 162 (2021). https://doi.org/10.1186/s42269-021-00612-z

15. Furia FF, Godfrey E, Mwamanenge N, Swai P. Spectrum of paediatric rheumatic disorders at a tertiary hospital in Tanzania. Pediatric Rheumatology. 2020 Dec; 18:1-8.

16. Godfrey EK, Furumbe EG, Faustine F, Naburi H. Collodion baby treated at a tertiary hospital in Tanzania: a case report. J Med Case Rep. 2018 Dec; 12:1-5.

17. Kiputa M, Urio O, Maghembe A, Kombo D, Dhalla S, Ndembo V, Muze K, Kahwa M, Fakih Z, Kija E. Confirmed diagnosis of classic Wiskott–Aldrich syndrome in East Africa: a case report. J Med Case Rep. 2022 Jul 27; 16(1):301.

18. Rohani-Montez SC, Bomberger J, Zhang C, Cohen J, McKay L, Evans WR. Educational needs in diagnosing rare diseases: A multinational, multispecialty clinician survey. Genet Med Open. 2023 Jan 1; 1(1):100808.

19. Willmen T, Willmen L, Pankow A, Ronicke S, Gabriel H, Wagner AD. Rare diseases: why is a rapid referral to an expert center so important?. BMC Health Serv Res. 2023 Aug 23; 23(1):904.

20. Rodrigues G, Poletto E, Baldo G. Basic and translational research in rare diseases in low-and middle-income countries: challenges and solutions. J Community Genet. 2024 Dec 13:1-3.

21. Flores A, Burgos S, Abarca-Barriga H. Knowledge level of medical students and physicians about rare diseases in Lima, Peru. Intractable Rare Dis Res. 2022 Nov; 11(4):180-188. doi: 10.5582/irdr.2022.01079. PMID: 36457581; PMCID: PMC9709623.

22. Requena-Fernández MA, Dasí F, Castillo S, Barajas-Cenobi R, Navarro-García MM, Escribano A: Knowledge of Rare Respiratory Diseases among Pediatricians and Medical School Students. J. Clin. Med. 2020, 9, 869; doi:10.3390/jcm9030869

23. Bavisetty S, Grody WW, Yazdani S. Emergence of pediatric rare diseases: Review of present policies and opportunities for improvement. Rare Diseases 2013; 1:e23579

24. Evans WR, Rafi I. Rare diseases in general practice: recognising the zebras among the horses. Br J Gen Pract. 2016 Nov; 66(652):550-551. doi: 10.3399/bjgp16X687625. PMID: 27789486; PMCID: PMC5072891.

25. Bolz-Johnson M, Clément L, Gahl W, Padilla C, Nishumura Y, Yang R, Sarfaty L, Hoogerbrugge N, Baynam G, Kenny10 T. Enhancing the value of clinical networks for rare diseases. Rare Dis Orphan Drugs J. 2022; 2(2):9.

26. Witt S, Schuett K, Wiegand-Grefe S. et al. Living with a rare disease - experiences and needs in pediatric patients and their parents. Orphanet J Rare Dis 18, 242 (2023). https://doi.org/10.1186/s13023-023-02837-9

27. Currie G, Szabo J: “It is like a jungle gym, and everything is under construction”: The parent's perspective of caring for a child with a rare disease. Child Care Health Dev. 2018; 1–8.

28. Gunne E, McGarvey C, Hamilton K, Treacy E, Lambert DM, Lynch SA. A retrospective review of the contribution of rare diseases to paediatric mortality in Ireland. Orphanet J Rare Dis. 2020 Nov 4; 15(1):311. doi: 10.1186/s13023-020-01574-7. PMID: 33148291; PMCID: PMC7641805.

Supporting information legends:

Dataset: This is the complete dataset of the findings of the study

Ethics approval: This is the ethics approval for the study from the institutional IRB

Data collection tool: This is the questionnaire that was administered via an online link to participants

Questionnaire first section: This shows the introductory section of the online questionnaire indicating the information on benefits, risks and confidentiality

Figure 3

Click here to access/download;Figure;Figure3.tif

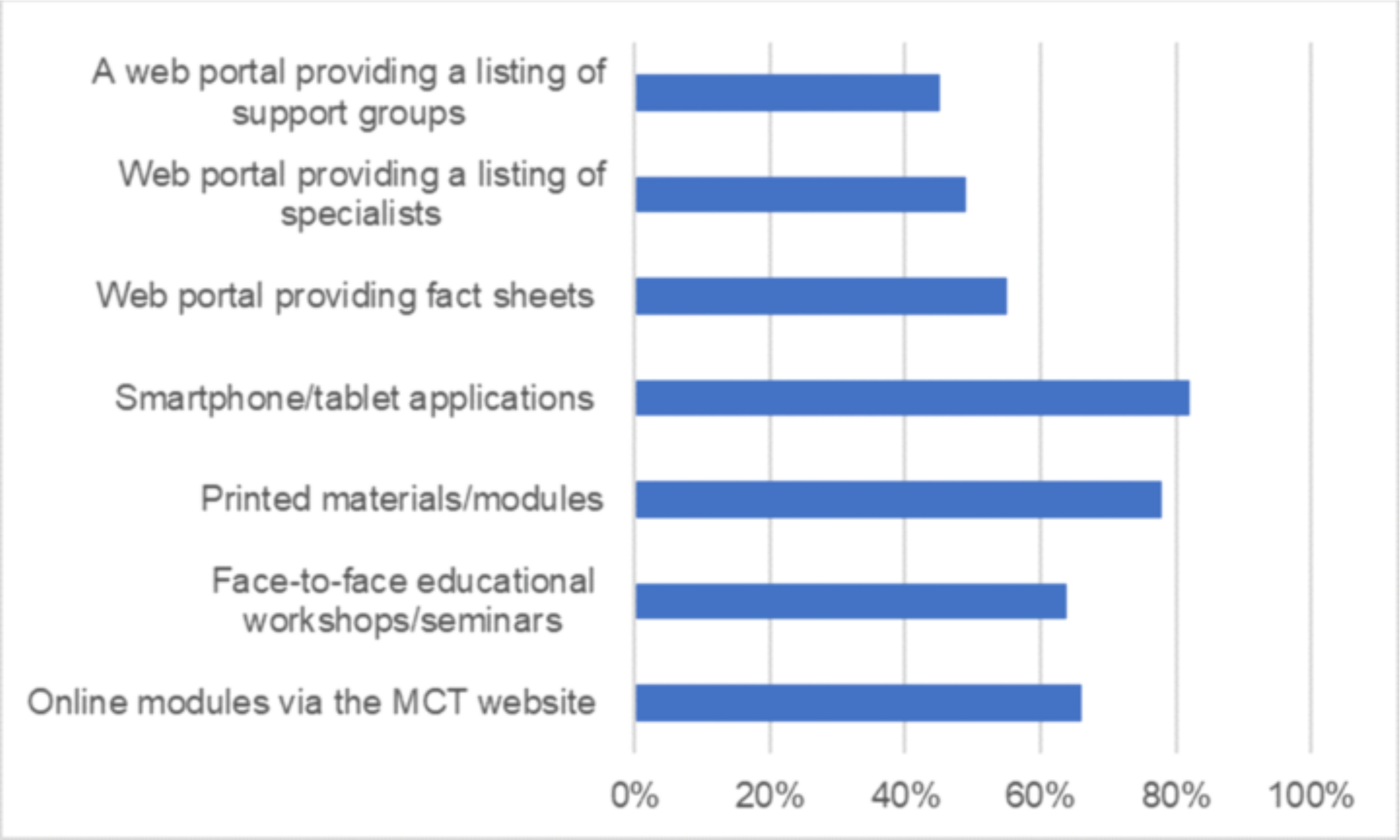

Figure 4

Click here to access/download;Figure;Figure4.tif

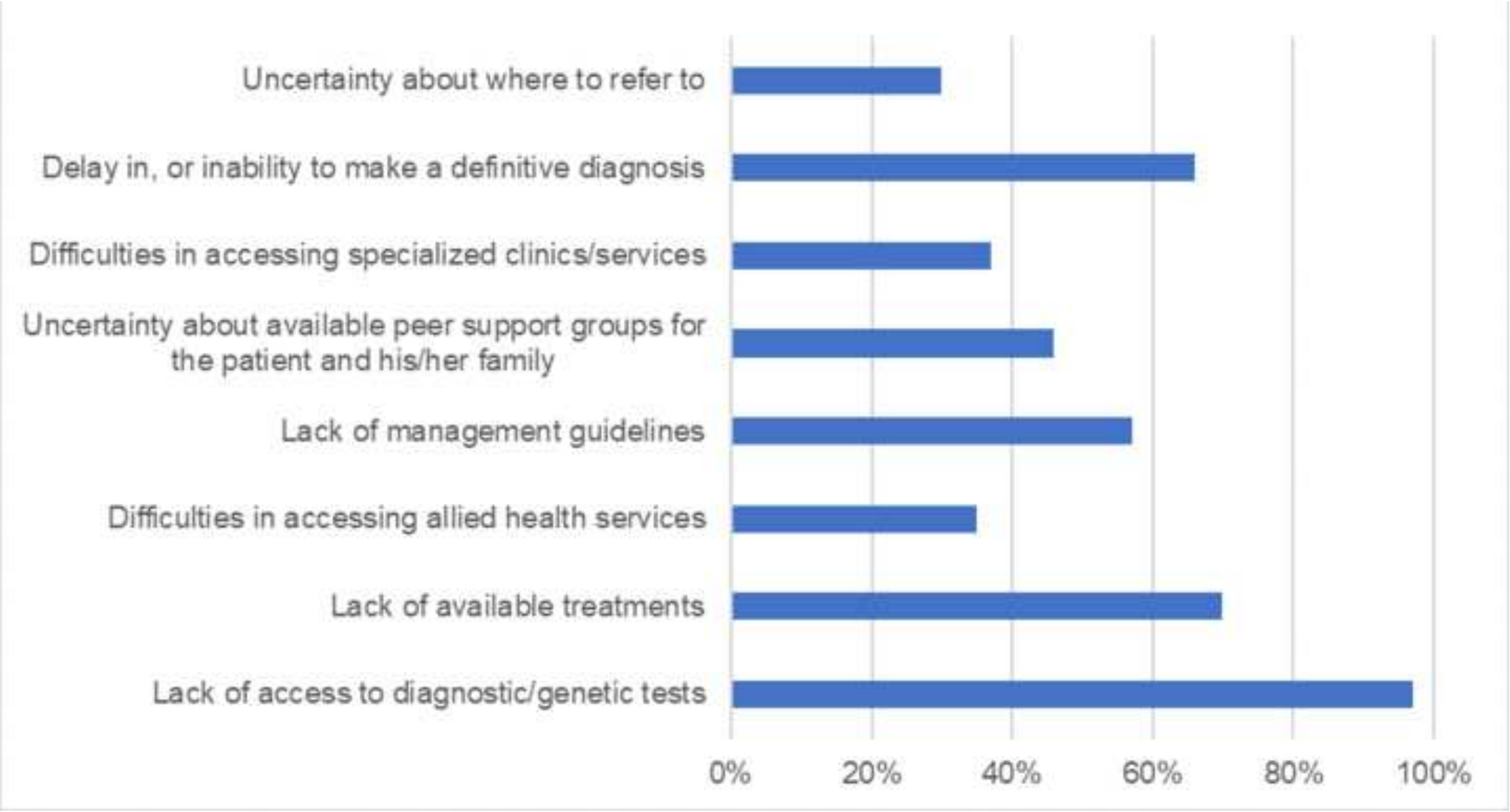

Figure 1

Click here to access/download;Figure;Figure1.tif

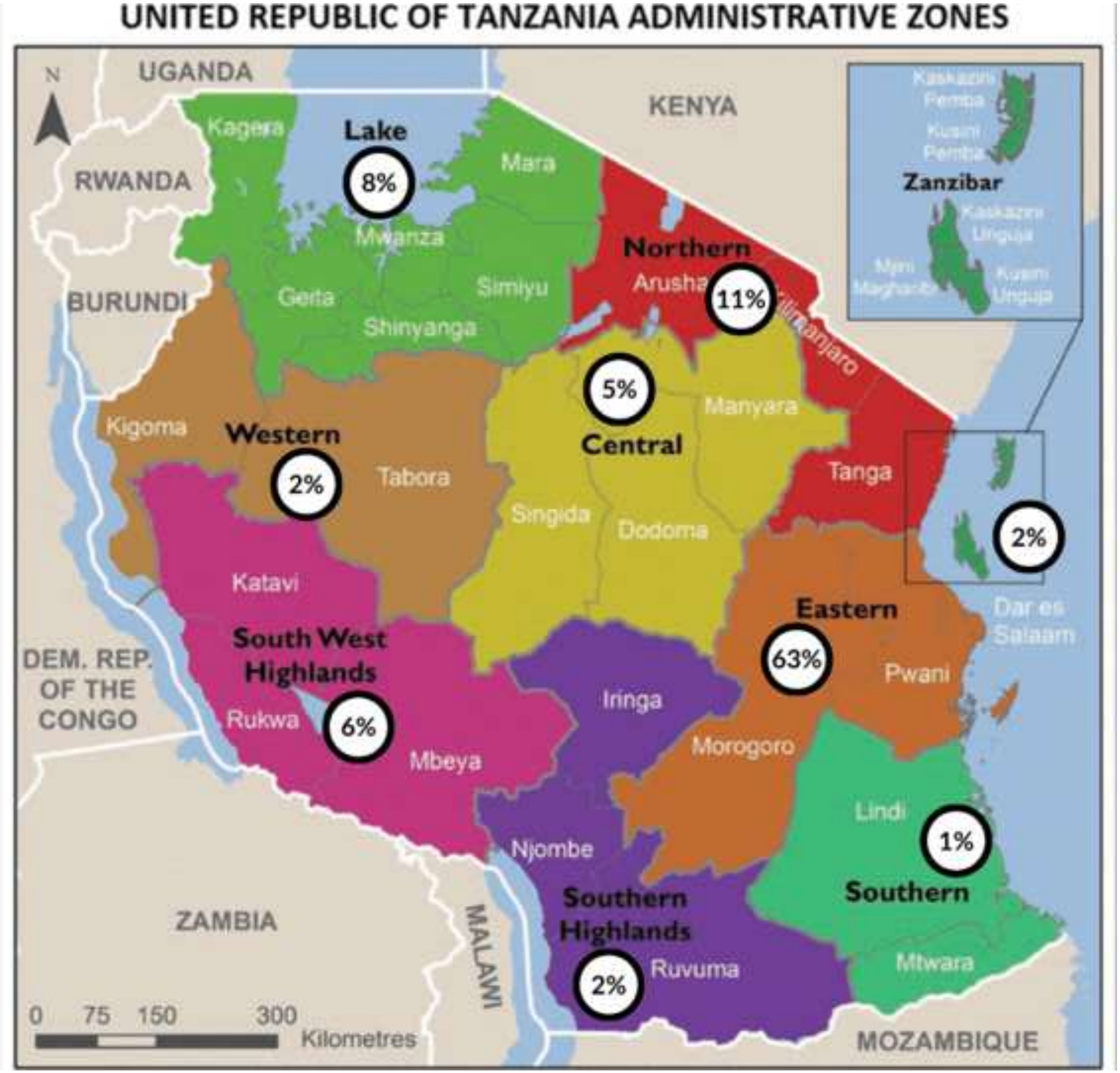

Figure 2

Click here to access/download;Figure;Figure2.tif

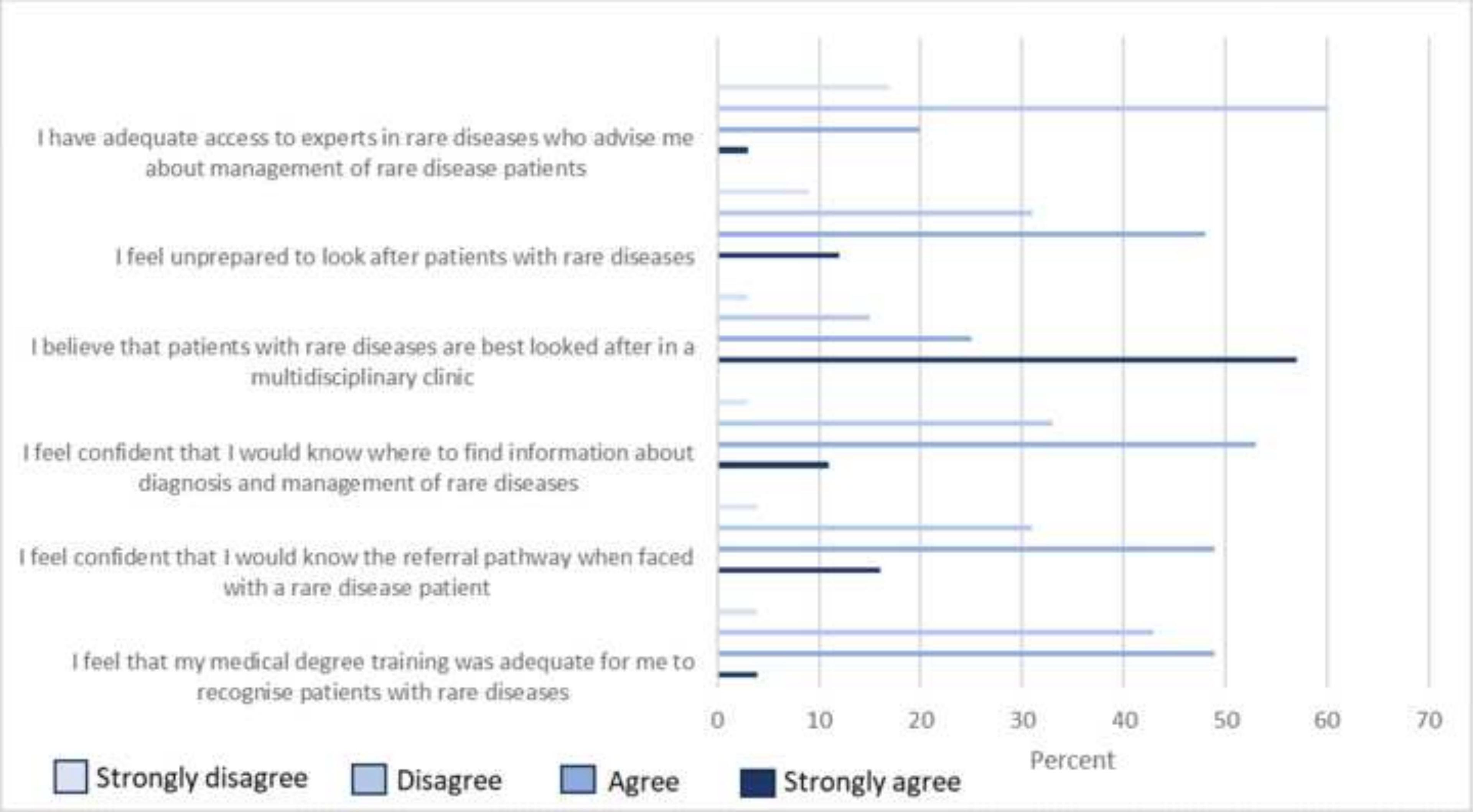

dataset

Click here to access/download
Supporting Information
FINAL DATA SET.csv

ethics approval

Click here to access/download
Supporting Information
Rare Disease KP- Approval.pdf

data collection tool

Click here to access/download
Supporting Information
Survey Tool_Rare Diseases Tz.pdf

Introductory section of questionnaire

Click here to access/download
Supporting Information
introductory section of questionaire.tif

Rare diseases in children - knowledge, experiences and challenges faced by Pediatricians in Tanzania

Mariam Noorani[1,2*], Mohamedraza Ebrahim [1], Francis Furia [2]

[1]Aga Khan University, Department of Pediatrics, Dar es Salaam, Tanzania

[2]Muhimbili University of Health and Allied Sciences, Department of Pediatrics and Child Health, Dar es Salaam, Tanzania.

*** Corresponding author**

email: mariam.noorani@aku.edu (MN)

All authors contributed equally to this work

Keywords: rare diseases, children, pediatricians, Tanzania, challenges, knowledge, experiences.

Abstract

Diagnosing and treating rare diseases in children is a major challenge for pediatricians across the world. There is a lack of adequate knowledge of these conditions and diagnostic testing is not easily accessible, which frequently results in delays in care. The knowledge, experiences and challenges faced by pediatricians in Tanzania is not known. This study presents the results of a nationwide cross-sectional survey which aimed to describe the knowledge of pediatricians in Tanzania on rare diseases, their experiences, and the challenges they face in treating these children. Of the 323 pediatricians registered on the Pediatric Association of Tanzania WhatsApp group, which is about 90% of pediatricians registered to practice in Tanzania, 168 completed the survey, giving a response rate of 52%. All of them had encountered a child with rare diseases in their career, with 60% having seen one in the 6 months preceding the survey. The commonest rare condition encountered was genetic/metabolic, and the most common difficulty (97%) encountered was lack of access to diagnostic testing. Only a third

of respondents reported that rare diseases were taught in university and 60% of felt unprepared to look after these children. Three quarter of respondents did not have access to experts to advise them on management. Rare diseases are commonly encountered by pediatricians in Tanzania, and there is a challenge in diagnostic testing as well as a gap in training, with pediatricians not confident in providing care. They also lack access to experts who can advise on rare disease management. There is an urgent need to find ways to make diagnostic testing available, accessible and affordable. A review of medical training curricula should be done to incorporate rare disease education and skill development. Strategies to connect pediatricians with regional and global experts should be put in place. The study findings may be limited by the low response rate, the use of technology to distribute the survey and imbalance of sub-specialty and general practice.

Introduction

Rare diseases are a group of medical conditions that affect a small number of people in the population. Definitions of rare diseases vary globally, and different criteria are used to describe these conditions. In the United States, a condition affecting less than 200,000 people is considered rare, while in Europe rare disease is one affecting less than 5 in 10,000 people. [1]

Globally, there are between 5000 to 8000 rare diseases that have been identified affecting between 350 and 475 million people. The burden of these conditions is presumed to be higher in African countries, which have limited capacity to diagnose these patients, as well as a lack of treatment options and high cost of care, resulting in economic hardships, severe complications, and subsequent disabilities and mortality. [2]

The diagnosis of these conditions depends largely on laboratory and genetic testing because of limited clinical experience with their manifestations, resulting in delayed diagnosis and treatment. Lack of

treatment guidelines and the rarity of expert clinicians with experience managing these conditions further compounds the problem. [3].

In developed countries, Paediatric sub-specialists, including geneticists, hematologists and endocrinologists, provide care to children with rare diseases. However, in low and middle income settings, primary care providers, including medical officers and pediatricians are on the front line of care for affected children [4]. Medical officers and pediatricians in low resource settings communicate with parents regarding diagnosis and potential management and coordinate referral to tertiary facilities for advanced management. Therefore, they need to have adequate knowledge to provide counselling, diagnostic information and care for families, particularly for conditions whose therapy is not accessible or not yet developed.

However, global data suggest that pediatricians and other specialist doctors are not adequately trained to recognize, diagnose, or manage these conditions. A nationwide survey of pediatricians from Australia showed that only about 50% of them reported that rare diseases were adequately covered during their training, while about a quarter felt unprepared to care for these children. [5] Similarly, over 90% of physicians undertaking specialization in Poland perceived their knowledge on rare diseases as poor and less than 5% felt confident of taking care of these patients. [6] A study from Spain found that less than a third of physicians reported receiving training on rare diseases during their undergraduate and postgraduate years. [7]

Very few studies gauging healthcare provider knowledge are available from the developing world. One survey from India reported that only a third of physicians surveyed had some knowledge of rare diseases while almost 80% reported they had no resources to diagnose and manage these conditions and their institution had no continuing education program in rare diseases. [8]

Apart from lack of adequate knowledge, there are many challenges that pediatricians face in the diagnosis and management of rare diseases. Studies from high-income countries, including Australia, have identified diagnostic delays, lack of available treatments, and uncertainty of where to refer as common challenges. [5] Lack of specialists was highlighted as a challenge in in Malaysia by Shafie et al, reporting only 13 rare disease specialists in the country. This study also showed that about 40% of patients with rare diseases were on symptomatic treatment only. [9]

Various interventions have been proposed to improve access to knowledge and improve the diagnosis of rare diseases. Physicians in a Belgian study recommended specific education on “red flags” or pointers to increase attentiveness to rare diseases. [10] Use of specific platforms like Orphanet and other rare disease online resources is another potential way to improve knowledge and its application towards timely identification of rare diseases.

In recent years, patient advocacy groups have accelerated the global progress in diagnosing and managing rare conditions by bringing together governments, patients and scientific communities. [3] The coming together of researchers and clinicians to form consortia has played a major role in furthering research and treatment of rare diseases, and pediatricians should be an integral part of such consortia.

In Tanzania, information is scarce on rare diseases in children; the epidemiology, the types and the management options are not known. Since 2016, the country has been commemorating the World Rare Disease Day, which has been driven by a patient advocacy group called Ali Kimara Rare Disease Foundation (AKRDF). This group brings together families affected by rare diseases and well-wishers from different sectors including media and sports who raise awareness on the plight of families. The rare disease agenda in the country has further been driven by the formation of the Tanzania Human Genetics Organization (THGO) in 2019 which aims to bring together various stakeholders in the field

of genetics. [11] These include geneticists, lab scientists, pharma experts and doctors who provide technical expertise, undertake research and advise communities and government on policy related to rare diseases.

In 2020, a national call to action was made during the commemoration of World Rare Disease Day that was co- hosted by the THGO and AKRDF. The call highlighted various areas of urgent action including identifying demographics of patients with rare diseases, developing guidelines for diagnostics and treatment as well as strengthening research. [12]

The rare occurrence of these diseases, the lack of epidemiologic data and unavailability of diagnostic testing and treatment guidelines may limit pediatricians in their ability to identify these patients. Many of these conditions, especially in the perinatal period present with signs and symptoms that may mimic other conditions. [13]

In Tanzania, there is no data on how frequently pediatricians encounter rare diseases in their practice, their knowledge of these conditions and the challenges that they face in management.

This study aimed to determine the knowledge, experiences and challenges perceived by pediatricians in Tanzania in managing presumed rare diseases. This is the first step in highlighting the burden of the problem. The data from this study will help to identify specific needs for training to improve knowledge and skills of pediatricians in managing children with rare diseases. Additionally, the challenges cited will prioritize areas for improvement. The data can also be used to inform stakeholders in medical education to include conditions from this group in the curriculum for medical training.

Materials and Methods

Study design, setting and population: This was a nationwide cross-sectional survey conducted among pediatricians working in Tanzania (Mainland and Zanzibar) who are members of Paediatric Association of Tanzania (PAT) and were included in the official PAT WhatsApp social media platform. PAT is the official professional association operating in United Republic of Tanzania, it had 325 members at the time of conducting this study (informal communication with PAT leadership), who were licensed and practicing at all levels of the health-care system from primary health care to tertiary referral facilities. The PAT WhatsApp platform is utilized for communication among members for social, academic and clinical issues. Members of the group are strictly pediatricians and are added soon after graduation. Over 90% of the pediatricians in the country are part of the group; those who are not are either non-practicing or choose to stay away from social media. The platform is also used for seeking consultation and discussion of cases managed by pediatricians who are in the group, discussions about patients are conducted with strict adherence to ethical principles of not sharing information that could reveal patients' identities. We used the WhatsApp group to disseminate this survey since it provided the widest reach to the target population.

Data collection instruments: Data was collected using a questionnaire (attached as supporting information) which was adapted from the instrument used in a similar study conducted in Australia. [5] This tool was found to be appropriate for this study because it provided comprehensive questions that aligned with the objectives of our research, making it a suitable foundation for adaptation. Questions were modified to fit the Tanzanian healthcare context; modifications mainly involved changes in geographical regions, regulatory authorities and access to relevant health care information. The modified questionnaire included sections on:

1. Pediatricians' demographics and practice which asked questions about years of experience, site of practice and sub-specialty training

2. Pediatricians' experience with rare disease patients including types of rare diseases, how many patients they had seen and how frequently they manage them
3. Challenges encountered by pediatricians in managing children with rare diseases.
4. Pediatricians' education, training and their self-reported knowledge of rare diseases which asked about rare disease education during undergraduate and postgraduate training as well as workshops or seminars related to rare diseases
5. Likelihood of use of resources which included questions about their preferred resource with information about rare diseases
6. Pediatricians' perceptions and confidence regarding providing care to children with rare diseases which asked about access to experts, knowing the pathway to channel a child with rare disease and their preparedness to provide care

Data collection process: The questionnaire was pilot-tested on 4 senior paediatric residents (postgraduate trainees) to ensure the meaning, content and flow of the questions was clear. We selected senior paediatric residents because they were almost graduating and would have relevant experience in understanding concepts and questions related to rare diseases. The online link was shared with them, they reviewed it, filled out all the sections and gave written feedback on each section of the questionnaire. Adjustments that were made based on their input included the flow and structure of the questions and additions to the list of available resources.

The questionnaire was then shared as an online link circulated to members in the WhatsApp platform. To encourage participation in the survey, zonal PAT representatives were informed about the survey and requested to remind their members to accept the link and respond to the survey. All pediatricians on the group were eligible to take the survey as long as they were in active clinical practice, this was indicated at the beginning of the online questionnaire.

Data handling and analysis: Data was collected between 7th January 2024 and 4th February 2024 and was auto-populated onto a Microsoft online Excel sheet. The data were checked for completeness and consistency and subsequently coded and analyzed using inbuilt formulae in Excel. Missing data in specific sections was noted and was omitted during analysis. Data were summarized into frequency distribution tables and charts that described the responses of participants regarding various aspects of rare diseases.

Ethical considerations: Ethical clearance was obtained from the Muhimbili University of Health and Allied Sciences (MUHAS) Institutional Review Board (ref: DA. 282/298/01.C/1895) and the permission to approach pediatricians was obtained from the professional society: Pediatric Association of Tanzania (PAT). The introductory section of the questionnaire provided information related to confidentiality, risks and benefits (Image provided as supporting information). Written consent was not obtained but was implied if the pediatrician filled out the online form and submitted it after reviewing the information provided.

Confidentiality was maintained by not collecting data on any direct identifiers like names or email addresses. Study data was kept confidential and accessible only to the investigating team by use of password protected computers.

Results

A total of 168 pediatricians completed the survey giving a response rate of about 52%. There was representation from all zones in the country with the majority (63%) coming from the Eastern zone where the commercial capital city – Dar es Salaam is located. Figure 1 shows the distribution of respondents from different zones of the country which is in keeping with the distribution of pediatricians in these areas

Fig 1. Distribution of respondents from different zones in Tanzania.

The map illustrates percentage of respondents from the different geographical zones in Tanzania. Map modified from Mzee, 2021 [14] by adding percentages. Available from: https://doi.org/10.1186/s42269-021-00612-z. Available via cc by 4.0 licence: http://creativecommons.org/licenses/by/4.0/.

Most participants (77%) were women and 79% were under the age of 45 years. Only about 30% had more than 10 years of clinical experience in the field of pediatrics. About half of the respondents worked in both public and private health care settings and the majority (81%) were general pediatricians with only a fifth being sub-specialized in a specific field of pediatrics. The characteristics of the participants are summarized in Table 1.

Table 1: Characteristics of study participants

VARIABLE	N (%)
Gender	
Male	39 (23%)
Female	129 (77%)
Age groups	
<= 45 years	132 (79%)
46-60 years	31 (18%)
>60 years	5 (3%)
Years of experience as a pediatrician	
<5 years	60 (36%)
5 - 10 years	52 (31%)
>10 years	53 (31%)
Missing data	3 (2%)
Type of practice	
Public sector only	30 (18%)
Private sector only	44 (26%)

Both public and private sector	94 (56%)
Specialty	
General pediatrics	136 (81%)
Pediatric Sub-specialty	32 (19%)

When asked how frequently rare diseases occur in Tanzania, most of the respondents (81%) believed that less than 5% of the Tanzanian child population is affected by rare diseases. Regarding their experiences in seeing children with rare diseases, all respondents had encountered a child with rare disease during their career but only 25 (15%) had seen more than 20 such children. Of these, about half of them were sub-specialists. Almost all (94%) reported looking after a child with an unusual cluster of symptoms and signs whose diagnosis could not be established and 22% reported having seen more than 10 such children. The most frequently encountered rare conditions were metabolic/genetic (74%) followed by neurological, musculoskeletal and cardiac. Table 2 summarizes the experiences in encountering rare diseases.

Table 2: Experiences of pediatricians in encountering rare diseases

**VARIABLE**	**N (%)**
Number of patients looked after with diagnosed rare diseases throughout career	
1-5 patients	88 (52%)
6-10 patients	38 (23%)
11-20 patients	17 (10%)
>20 patients	25 (15%)
The number of patients looked after without definitive diagnosis reached	
1-5 patients	94 (56%)
6-10 patients	36 (22%)
11-20 patients	19 (11%)
>20 patients	19 (11%)

Types of rare conditions encountered[1]	
Metabolic/genetic	124 (74%)
Neurological	90 (54%)
Musculoskeletal	59 (35%)
Cardiac	59 (35%)
Endocrine	55 (33%)
Immunological	53 (32%)
Dermatological	54 (32%)
Gastrointestinal	47 (28%)
Respiratory	46 (27%)
Cancer	45 (27%)
Connective tissue	42 (25%)
Renal	38 (23%)
Rheumatic	33 (20%)
Infectious	26 (16%)
Hematologic	2 (1%)
When the most recent suspected or diagnosed rare condition was seen	
< 6 months ago	101 (60%)
6-12 months ago	46 (27%)
1-3 years ago	12 (7%)
>3 years ago	9 (6%)

[1] Multiple selection was allowed for this question

When asked about challenges encountered while managing children with rare diseases, the commonest difficulty was lack of access to diagnostic and genetic tests with 97% of pediatricians encountering this difficulty. Other common challenges encountered included a lack of treatments (70%) and delay or inability to make a definitive diagnosis (66%). The challenges encountered are summarized in figure 2.

Figure 2. Challenges encountered by pediatricians in managing rare diseases

Regarding training received on rare diseases, only about a third of respondents said rare diseases were taught during their undergraduate curriculum and a similar proportion reported being taught during their postgraduate training. Only 30% of them had ever attended any workshops or lectures related to rare diseases.

On exploring their perceptions and confidence in recognizing and managing rare diseases, only about half of the respondents felt that their training was adequate for them to recognize patients with rare diseases and about 65% felt confident that they would know the referral pathway for a child with rare disease. About two-thirds (60%) felt unprepared to look after patients with rare diseases and over 70% did not have access to experts to support them in providing care. Responses to other sections of the questionnaire including confidence in finding rare disease information and perception on role of multidisciplinary care are further described in Figure 3.

Figure 3. Perception and confidence in caring for children with rare diseases

When asked about the likelihood of using resources with information on rare diseases, over 80% of respondents favored a smartphone or tablet application while about 60% would use online modules via the Medical Council of Tanzania (MCT) website and a similar percentage would utilize face-to-face workshops or seminars. The likelihood of resource use by respondents are summarized in figure 4.

Figure 4. Likelihood of resource utilization of pediatricians managing rare diseases

Discussion

This study was conducted to determine knowledge, experiences and challenges in managing rare diseases among pediatricians in Tanzania. To the best of our knowledge, this is the first research study to investigate the experience of health care providers in managing rare diseases in Tanzania, the challenges faced in their treatment and the education and training about these conditions. There have been case reports and case series from Tanzania on individual rare diseases or rare diseases related to

specific systems like rheumatology [15~~4~~-17~~6~~], however the overall spectrum of rare diseases has not been described. The findings of our study, having focused on pediatricians who are the specialists most likely to encounter rare conditions, can act as a proxy of the situation of rare disease knowledge, experiences and challenges in Tanzania.

All participants in our study reported having managed rare disease patients during their career; however, only 25% of participants had managed more than 10 children with rare diseases. These findings reflect on the overall prevalence of these conditions; individually they may be rare, but as a group they are encountered in clinical practice although infrequently; similar to the findings by Rohani-Montez et al in a study conducted among clinicians in Europe and the United States where more than half reported that they have rarely (1-2 patients per year) seen patients with a rare disease. [18~~7~~]

It is plausible to attribute the reported experience of managing a higher number of children to sub-specialty training; 19% of participants had sub-specialty training while 25% of participants had managed more than 10 children with rare disease. Healthcare providers with sub-specialty and /or multiple specialties have better knowledge and experience in managing children with rare diseases and are usually practicing in referral and specialized healthcare facilities. [19~~8~~]

Limited access to diagnostic testing was the challenge most commonly faced by the participants in this study. This is because of lack of availability of genetic testing in Tanzania and the added high cost of testing if the sample is sent outside the country. The difficulties in accessing genetic testing has been highlighted as a problem even in high income countries as reported by Zurynski in Australia. [5] Additionally, pediatricians in Australia also reported delays in interpretation of genetic tests; highlighting that apart from the physical diagnostic infrastructure, equally important is the human resource of clinical geneticists. In Tanzania, clinical genetics is a specialization that is not readily available with only 1 specialist in the country.

Comparative data from other low- and middle-income countries (LMICs) highlight similar challenges in the diagnosis and management of rare diseases in children. Studies conducted in LMICs indicate that pediatricians in these regions face significant barriers, including limited access to diagnostic tools, a lack of specialized expertise, and inadequate training on rare diseases—findings that closely align with our study in Tanzania. For instance, research from sub-Saharan Africa and Southeast Asia has noted the scarcity of genomic sequencing availability and the higher costs associated with diagnostic testing, contributing to delayed diagnosis and treatment. [20~~19~~]

Pediatricians in this study expressed a lack of training on rare diseases in both undergraduate and postgraduate training. There was also poor confidence in diagnosing and managing children with rare diseases with the majority indicating a lack of confidence. Similar findings have been documented from other studies globally, indicating low knowledge and ability to diagnose patients; this has consequently resulted in delays in diagnosis and treatment. [21~~0~~-22~~1~~] There is also lack of awareness of the global epidemiology with most pediatricians believing that less than 5% of Tanzanian children are affected by rare diseases; which is lower than the estimated global prevalence of rare diseases of between 6-8%.

Capacity building for managing children with rare diseases in the form of guidelines, treatment, and care pathways with improvement in diagnostic facilities including treatment networks have been advocated for in several studies. [23~~2~~, 24~~3~~] Participants in our study suggested various methods that can be used to improve their knowledge and skills on rare diseases. Use of a smart phone application with all relevant information on rare diseases is the resource most favored by pediatricians in Tanzania. This is likely due to the current age of digitalization and familiarity with technology. Majority of our study participants were less than 45 years of age and this is a generation which is comfortable and confident in using smart phone applications.

Other resource options include physical and virtual workshops, and availability of materials through web portals. Similar approaches were reported in an online survey which was conducted among clinicians in Europe and the United States who preferred a comprehensive online learning platform with current education on rare diseases. [17] It is therefore imperative for stakeholders involved in the care of these children including patient support groups, paediatric associations, medical schools, hospitals and the Ministry of Health to strategize and organize coordinated educational programs.

Most participants in this study agreed that children with rare disease should be managed by a multidisciplinary team, however less than a quarter of them had access to experts who could advise on the management of these patients. This shows a possible gap in coordinated care at different levels of the health system but could also be a reflection of lack of specialized pediatricians with the required knowledge and skills to assess and manage rare diseases. Globally, there has been a move to develop rare disease clinical networks that span across continents and harness the expertise of clinicians and researchers to provide the best possible care to patients. [254]

Rare diseases cause significant burden to the patients, families, communities and health systems. Diagnostic delay is costly to the families and health systems and this is worsened by the poor knowledge and skills of practitioners. [18, 265, 276] These diseases contribute significantly to morbidity and mortality among children including infants and neonates, [287] it is therefore important to consider strategies for mitigating existing challenges with a priority on capacity building for healthcare providers.

Our study has some limitations to note. Firstly, the response rate was 52% hence may not be reflective of all pediatricians in Tanzania. Secondly, given that a significant proportion (79%) of respondents were under 45 years of age, there is a possibility that the study may be biased toward younger pediatricians. Thirdly, among the respondents, pediatricians with sub-specialty training were 19% and they are more likely to recognize and manage rare disease compare to general pediatricians hence this

may bias the findings. Finally, the use of an online survey via the PAT WhatsApp group may have excluded some eligible participants who were not on the group, as well as those who were not familiar or comfortable with the use of technology to complete online surveys.

To address these limitations, future research could aim for a more representative sample, incorporating strategies to enhance participation from older pediatricians. Additionally, further studies could explore whether digital tools are equally acceptable across different age groups. Despite these limitations, the study provides valuable insights into the challenges faced by pediatricians regarding rare diseases and highlights a significant gap in training and diagnostic resources.

Conclusions and Recommendations

Our findings suggest that pediatricians in Tanzania frequently encounter children with presumed rare diseases, with genetic/metabolic conditions being the most common from their perspective. However, a significant challenge lies in the limited access to diagnostic testing and lack of experts, which hinders effective management of these conditions. Furthermore, there is a notable gap in the training on rare diseases, leaving many pediatricians feeling unprepared to care for these children.

To improve the care of children with rare diseases in Tanzania, there is urgent need to improve access to diagnostic testing and reduce the cost by providing subsidies and creating mechanisms through which these tests can be easily requested when needed.

A review of undergraduate and postgraduate medical curricula should be done to incorporate rare diseases as an important component of training. Access to relevant expertise and resources should be prioritized and networks of care developed to connect pediatricians with national, regional and global experts.

We recommend that future studies should focus on developing a registry to document the true spectrum of rare diseases in Tanzania. Population based studies should be conducted to quantify the prevalence

and burden of rare diseases and genetic and genomic research should be encouraged for accurate identification and risk stratification. Exploring the experiences of families of children with rare diseases is equally important to identify their challenges and needs for better support systems.

Acknowledgments

The authors acknowledge the support from the Paediatric Association of Tanzania in conducting this study.

References

1. Richter, T., Nestler-Parr, S., Babela, R., Khan, Z. M., Tesoro, T., Molsen, E., & Hughes, D. A. (2015). Rare Disease Terminology and Definitions—A Systematic Global Review: Report of the ISPOR Rare Disease Special Interest Group. Value in Health, 18(6), 906–914. https://doi.org/10.1016/j.jval.2015.05.008

2. Adachi T, El-Hattab AW, Jain R, Nogales Crespo KA, Quirland Lazo CI, Scarpa M, Summar M, Wattanasirichaigoon D. Enhancing equitable access to rare disease diagnosis and treatment around the world: a review of evidence, policies, and challenges. Int J Environ Res Public Health. 2023 Mar 8; 20(6):4732.

3. Stoller J. K. The Challenge of Rare Diseases. CHEST. 2018; 153(6), 1309–1314.

4. Baynam, G., Hartman, A. L., Letinturier, M. C. v, Bolz-Johnson, M., Carrion, P., Grady, A. C., Dong, X., Dooms, M., Dreyer, L., Graessner, H., Granados, A., Groza, T., Houwink, E., Jamuar, S. S., Vasquez-Loarte, T., Tumiene, B., Wiafe, S. A., Bjornson-Pennell, H., & Groft, S. (2024). Global health for rare diseases through primary care. The Lancet Global Health, 12(7), e1192–e1199. https://doi.org/10.1016/S2214-109X(24)00134-7

5. Zurynski Y, Gonzalez A, Deverell M, Phu A, Leonard H, Christodoulou J, & Elliott E. Rare disease: a national survey of pediatricians' experiences and needs. BMJ Paediatr Open. 2017; 1(1). https://doi.org/10.1136/BMJPO-2017-000172

6. Walkowiak D, & Domaradzki J. Are rare diseases overlooked by medical education? Awareness of rare diseases among physicians in Poland: an explanatory study. Orphanet J Rare Dis. 2021; 16(1), 1–12.

7. Ramalle-Gómara E., Domínguez-Garrido E, Gómez-Eguílaz M, Marzo-Sola M. E, Ramón-Trapero J. L & Gil-De-Gómez J. Education and information needs for physicians about rare diseases in Spain. Orphanet J Rare Dis. 2020; 15(1), 1–7.

8. Agrawal R, Amaresh Rao M, Brian M, Chowdary G, Gayatri K, Krishnaji Rao M. Baseline knowledge of rare diseases in India—a survey. Int J Rare Dis Disord. 2019; 2(1):4-11.

9. Shafie A. A, Supian A., Ahmad Hassali M. A, Ngu L. H, Thong M. K, Ayob H, & Chaiyakunapruk N. Rare disease in Malaysia: Challenges and solutions. PloS one. 2020; 15(4), e0230850. https://doi.org/10.1371/journal.pone.0230850

10. Vandeborne L, van Overbeeke E, Dooms M, de Beleyr B, & Huys I. Information needs of physicians regarding the diagnosis of rare diseases: A questionnaire-based study in Belgium. Orphanet J Rare Dis. 2019; 14(1), 1–11.

11. Alimohamed MZ, Mwakilili AD, Mbwanji K, Manji ZK, Kaywanga F, Mwaikono KS, Adolf I, Makani J, Hamel B, Masimirembwa C, Ishengoma DS. Inauguration of the Tanzania Society of Human Genetics: biomedical research in Tanzania with emphasis on human genetics and genomics. Am J Trop Med Hyg. 2021; Feb; 104(2):474.

12. Kaywanga F, Alimohamed MZ, David AB, Maeda D, Mbarak S, Mavura T, Nkya S, Ishengoma DS. Rare diseases in Tanzania: a National Call for Action to address policy and urgent needs of individuals with rare diseases. Orphanet J Rare Dis.2022. Sep 5; 17(1):343.

13. Bordini, B. J., & Kliegman, R. M. (2020). Undiagnosed and Rare Diseases in Perinatal Medicine: Uncommon Manifestations or Mimics of Neonatal Disorders. *Clinics in Perinatology*, *47*(1), xxi–xxii. https://doi.org/10.1016/j.clp.2019.12.001

14. Mzee, T., Kazimoto, T., Madata, J. et al. Prevalence, antimicrobial susceptibility and genotypic characteristics of Staphylococcus aureus in Tanzania: a systematic review. Bull Natl Res Cent 45, 162 (2021). https://doi.org/10.1186/s42269-021-00612-z

154. Furia FF, Godfrey E, Mwamanenge N, Swai P. Spectrum of paediatric rheumatic disorders at a tertiary hospital in Tanzania. Pediatric Rheumatology. 2020 Dec; 18:1-8.

165. Godfrey EK, Furumbe EG, Faustine F, Naburi H. Collodion baby treated at a tertiary hospital in Tanzania: a case report. J Med Case Rep. 2018 Dec; 12:1-5.

176. Kiputa M, Urio O, Maghembe A, Kombo D, Dhalla S, Ndembo V, Muze K, Kahwa M, Fakih Z, Kija E. Confirmed diagnosis of classic Wiskott–Aldrich syndrome in East Africa: a case report. J Med Case Rep. 2022 Jul 27; 16(1):301.

187. Rohani-Montez SC, Bomberger J, Zhang C, Cohen J, McKay L, Evans WR. Educational needs in diagnosing rare diseases: A multinational, multispecialty clinician survey. Genet Med Open. 2023 Jan 1; 1(1):100808.

198. Willmen T, Willmen L, Pankow A, Ronicke S, Gabriel H, Wagner AD. Rare diseases: why is a rapid referral to an expert center so important?. BMC Health Serv Res. 2023 Aug 23; 23(1):904.

2019. Rodrigues G, Poletto E, Baldo G. Basic and translational research in rare diseases in low-and middle-income countries: challenges and solutions. J Community Genet. 2024 Dec 13:1-3.

210. Flores A, Burgos S, Abarca-Barriga H. Knowledge level of medical students and physicians about rare diseases in Lima, Peru. Intractable Rare Dis Res. 2022 Nov; 11(4):180-188. doi: 10.5582/irdr.2022.01079. PMID: 36457581; PMCID: PMC9709623.

22~~1~~. Requena-Fernández MA, Dasí F, Castillo S, Barajas-Cenobi R, Navarro-García MM, Escribano A: Knowledge of Rare Respiratory Diseases among Pediatricians and Medical School Students. J. Clin. Med. 2020, 9, 869; doi:10.3390/jcm9030869

23~~2~~. Bavisetty S, Grody WW, Yazdani S. Emergence of pediatric rare diseases: Review of present policies and opportunities for improvement. Rare Diseases 2013; 1:e23579

24~~3~~. Evans WR, Rafi I. Rare diseases in general practice: recognising the zebras among the horses. Br J Gen Pract. 2016 Nov; 66(652):550-551. doi: 10.3399/bjgp16X687625. PMID: 27789486; PMCID: PMC5072891.

25~~4~~. Bolz-Johnson M, Clément L, Gahl W, Padilla C, Nishumura Y, Yang R, Sarfaty L, Hoogerbrugge N, Baynam G, Kenny10 T. Enhancing the value of clinical networks for rare diseases. Rare Dis Orphan Drugs J. 2022; 2(2):9.

26~~5~~. Witt S, Schuett K, Wiegand-Grefe S. et al. Living with a rare disease - experiences and needs in pediatric patients and their parents. Orphanet J Rare Dis 18, 242 (2023). https://doi.org/10.1186/s13023-023-02837-9

27~~6~~. Currie G, Szabo J: "It is like a jungle gym, and everything is under construction": The parent's perspective of caring for a child with a rare disease. Child Care Health Dev. 2018; 1–8.

28~~7~~. Gunne E, McGarvey C, Hamilton K, Treacy E, Lambert DM, Lynch SA. A retrospective review of the contribution of rare diseases to paediatric mortality in Ireland. Orphanet J Rare Dis. 2020 Nov 4; 15(1):311. doi: 10.1186/s13023-020-01574-7. PMID: 33148291; PMCID: PMC7641805.

Supporting information legends:

Dataset: This is the complete dataset of the findings of the study

Ethics approval: This is the ethics approval for the study from the institutional IRB

Data collection tool: This is the questionnaire that was administered via an online link to participants

Questionnaire first section: This shows the introductory section of the online questionnaire indicating the information on benefits, risks and confidentiality

RESPONSE TO REVIEWERS

COMMENT	RESPONSE
1. Confirm whether the data and text presented in this manuscript have not previously been published. Cover letter states that findings build upon previous published research. Clarification of this would be appreciated	We acknowledge the query and apologise for the confusing statement in the cover letter. What we meant is that our findings add to the existing knowledge on rare diseases which is predominantly from the global north. Ours now builds on this by sharing evidence from the global south. We have made edits to the cover letter to reflect this
2. Confirm whether this manuscript (or any portion thereof) is being considered for publication elsewhere	We confirm that this manuscript or any portion is NOT being considered elsewhere for publication. Have indicated this in the cover letter.
3. Use American English spelling.	This is noted and we have adjusted
1. Rephrase first sentence for clarity. Suggested to rephrase to something like this "Diagnosing and treating rare diseases in children remain major challenges for pediatricians across the world."	This suggestion is appreciated and has been incorporated
2. The second sentence state that the research gap is to understand how often Tanzanian pediatricians are met with rare diseases, yet the next sentence focuses the paper into other issues such as knowledge, challenges in treatment, etc. Which is the gap and the focus of this paper?	We thank the editor for highlighting this. We have edited to keep the focus on knowledge, practices and challenges
3. Fourth sentence, suggest replacing "This" with "This study presents the results of a..."	This suggestion is accepted and the sentence has been amended
4. How many total pediatricians are there in Tanzania? What proportion of those are in the WhatsApp group?	We have now included this information
4.a. - for the manuscript, not the abstract, make sure to discuss selection bias - how do pediatricians get added to the WhatsApp group? Who decides, and under what criteria, who is admitted into the WhatsApp group? Is it possible that a non-pediatrician is in the WhatsApp group? How do the pediatricians in the WhatsApp group differ from pediatricians outside the group? Why use WhatsApp group and no other more commonly used participant recruitment? - Some of this is already addressed in lines 100-107 but not all, make sure that all of this is adequately addressed.	This suggestion is appreciated and we have discussed it further in the methodology
5. for the manuscript, not the abstract, how do you reconcile that such a high proportion (88%) of pediatricians have encountered rare diseases	We acknowledge the query raised. Although these diseases are rare individually, together they are found in 6 – 8% of the population as

in their practice, when, by definition, rare diseases show extremely low frequency in either incidence or prevalence?	we mentioned in the introduction, this is in keeping with our findings, where majority of the respondents had encountered a suspected rare disease in their practice, showing that their overall incidence/prevalence is not that rare. This is now reflected in the discussion
6. For the manuscript, discuss how a phone app with information will meet the need of lack of access to diagnostic tests.	The phone app was the recommended resource that paediatricians favoured – above websites and print material to bridge the knowledge gap. This has now been reflected in the recommendations.
7. Discuss the limitations of the study in the abstract.	We have briefly discussed them
II. INTRODUCTION.<br>1. The citation offered for the definition of rare diseases does not link to a European definition of rare diseases. Either add a citation for the European definition OR move the citation to the right location in the sentence and add a citation for the European definition in the right location of the sentence. Also, please cite either peer-reviewed literature OR a widely accepted textbook on rare diseases for definition, as opposed to a website. See, for instance, https://doi.org/10.1016/j.jval.2015.05.008	We appreciate the suggestion and we have included the recommended citation which provides definitions used globally
2. Citation 2 (Adachi et al) contains information that is either contradictory or different or lacking to what is stated in the text (esp. regarding number of rare diseases and percentage of global population affected by it). Please update the information in the sentence OR add appropriate citation (if new citation is added, ensure that numbering is updated AND that the new citation is either more recent or more rigorous on its methodology than Adachi et al).	We acknowledge the discrepancy and have adjusted the text to reflect what is in the paper by Adachi
3. Sentences in lines 37-40 are missing citation. Please add.	Have modified the sentence and added the citation
4. Citation 3 in line 44 does not match the text of the sentence. Ensure that all citations match the sentence correctly.	We acknowledge the error and have changed the citation
5. Line 44, specify what "our setting" mean.	We have adjusted the wording to reflect what we mean
6. Rephrase line 61 for clarity.	We have rephrased this
7. Line 62, comma after "Australia" is missing.	Have now included the comma
8. Lines 62-66, explain how the information from these studies reflect conditions in which adequate knowledge exist OR put the information in the right context.	We have adjusted the initial line so that it no longer reflects on conditions of adequate knowledge

9. Line 68, replace "red flags" for appropriate medical term.	We appreciate the suggestion, however, we have used the terminology based on how the authors of the paper described it.
10. Lines 73-74, text almost verbatim from Stoller - please rewrite this paragraph.	We have rewritten as per the suggestion
11. Please specify who are the members of THGO. Try to contrast membership, mandate and activities of AKRDF and THGO for the reader to understand the context of the field in Tanzania better.	We have included these
12. Sentence ending in line 89 is missing citation.	We have added the citation
13. As mentioned above, clarify research gap and study aim AND ensure consistency.	We have amended to reflect the aims consistently
14. Line 95 - is it certain that this data WILL inform medical education? If not, then replace will by another appropriate modal verb or appropriate sentence.	This is acknowledged and we have adjusted it
III. MATERIALS AND METHODS<br>1. Add sub-headings. At minimum, Setting and Study Population, Sample Size and Sampling Methodology, Data Collection Instruments, Data Collection Process, Data Manipulation and Data Analysis, Ethical Considerations.	We have included relevant sub-headings as suggested
2. Line 98, delete "of" before "conducted."	Acknowledged and deleted
3. Line 99. Suggest to place "mainland and Zanzibar" in parenthesis.	Acknowledged and parenthesis added
4. Line 101, add citation for the number of members in PAT.	This was informal communication with PAT leadership and has been included as such
5. Line 105. Review "who are the group," it does not seem to make sense in this sentence.	The word “in” was missing and has been added
6. Line 105, do you mean "about" instead of "involving"?	Yes, and we have adjusted the wording
7. Line 109. Replace "in the survey done in Australia" for a more formal writing style.	We have replaced it for a more formal structure
8. Line 111. Explain the "modification" process employed to adapt the survey to the Tanzanian context.	We have briefly explained these modifications
8.a. what frameworks or theories guided this process?	The modification was mainly based on appropriate geographic, regulatory and resource related terminologies hence there was no specific frameworks or theories
9. Line 112. Does the "questionnaire" refer to the original (i.e., Australian) OR the modified (i.e., Tanzanian) context?	It refers to the modified one, we have now specified that
10. Lines 113-119. Ensure consistency of use between "participant" and "pediatrician" - stick to one or the other.	We acknowledge this and have modified to use pediatrician consistently
11. Line 120. Replace "pilot tested" for "pilot-tested."	Replaced

12. Line 120. Specify how many pediatricians participated in the pilot testing (and how were they selected).	We have included this
12.a explain in detail the process, methodology and theoretical foundation of the pilot testing process.	We have now explained this process. There was no specific theoretical foundation we used.
13. What was learned from the pilot testing? Did some question need to be further adjusted? What input was received from participants in the pilot testing? Explain here.	We have now explained this
14. Describe eligibility criteria.	We have added an explanation of this
14a. Were pilot testing participants allowed or not allowed to take part of the survey? Why or why not?	Pilot testing was done on senior paediatric trainees who were not eligible to participate in the survey
15. Line 125, replace "to" with "and" before "4th."	Replaced as suggested
16. What software was used to collect survey data?	We used Microsoft excel, this has been specified in the text
17. Line 126. How was incomplete data handled?	We have now explained this
18. Line 131. Clarify what is meant by "implied" informed consent. Were participants asked for consent as part of the online survey? Show a screenshot of the survey's first page.	We have further explained this and provided a screenshot as additional file.
19. Describe data protection and cybersecurity steps taken to ensure privacy and confidentiality of data.	We did not take any specific measures related to cybersecurity, however data was kept confidential by avoiding use of identifiers and being accessible only to the 3 study investigators.
IV. RESULTS<br>1. Line 138. Text states "most regions" - clarify how many regions (out of how many are represented).	We have adjusted this to reflect zones rather than regions as per the figure 1
2. Figure 1 shows % from regions, how does this compare to the % of PAT members per region?	It corresponds – we have included this
3. Line 139. The word "zones" is used whereas previously the word "regions" was used. Clarify and keep consistency.	We have clarified and used zones consistently
4. Line 145. Describe which fields of pediatric subspecialty are represented.	We did not ask them to specify the subspecialty field, the question asked them to choose between general paediatrics or sub-speciality paediatrics with a third option of other. In some cases, people populated other to describe their relevant sub-specialisation
4. Line 147 - Table 1. Add "missing" category for the "years of experience as a pediatrician" variable.	Added as advised
5. Line 148 - Table 1. Use formatting where only top and bottom lines for the table and for the	Formatted as per advice

headings is shown - eliminate lines in the middle (between rows and between columns) and the sides.	
6. Line 150. Contrast participants' beliefs (re: % of Tanzanian population affected by rare diseases) with reality.	This has now been added in the discussion
7. Line 153. Word "subspecialties" is used, whereas previously the word "specialties" (or derivatives) has been used. Recommend consistent use of "sub-specialties" (and derived words) across the document.	We have adjusted this throughout the document
8. Line 155. Better explained what is meant by "the commonest rare conditions."	We have adjusted the statement to say the most frequently encountered rather than commonest
9. Line 158 - Table 2. Format as suggested above.	Formatted
10. Line 158 - Table 2. "Number of patients ... without ... diagnosis" adds up to 99%. Please correct.	Adjusted
11. Line 158 - Table 2. "Types of ..." does not add up to 168 nor to 100. Explain with a footnote.	Explained
12. Line 158 - Table 2. "... most recent..." does not add to 100%. Please correct.	Corrected
13. Line 160. Specify 97% of what?	Specified
14. Line 167. How many participants have attended CME or specialty/sub-specialty conferences where they could have been exposed to appropriate rare diseases content? Was this explored?	WE have included the percentage that had attended conferences/training
15. Line 168. Specify, half of what?	Have specified
16. Line 170 and in general. Do not start sentences with numbers nor acronyms.	Have adjusted
17. Line 171. There is a reference to "domains" but domains explored have not been previously introduced. Explain domains (and theories the work draws from) in the methods section.	The wording has been changed to sections instead of domains – reflecting on how the questionnaire was organised
18. Figure 3 cannot be understood as is. Add appropriate contextual notes, legends, better categories, etc. to understand what information is being presented.	We have modified the figure to improve presentability
19. Line 174. Does this refer to currently available resources (i.e., apps or online	It refers to those they were most likely to use if available (whether or not they are currently

websites) or resources they wished they had but they do not have access to right now?	available). We have reworded the text to reflect this
20. Figure 3 does not match context described in text.	We have revised figure 3 to match the context
21. Figure 4 does not match context described in text.	Have replaced figure 4 with the correct figure
22. Need to better explain and connect survey to results. Need to add phrases like, "when asked about XXXX" participants showed "XXX"	We have rephrased the results as per the recommendation
22a. in the methods section describe the structure of the survey. For instance, Part 1 asked questions about XXXX such as "XXXXXX;" Part 2 asked XXXXXX, etc.	We appreciate the suggestion, and have explained in the methods
V. DISCUSSION.<br>1. Lines 180-181. Before the suggestion was made to clarify research gap and survey intent. Make sure that this line remains consistent with any changes made.	We have changed to maintain consistency
2. Lines 181-185. There is no need to restate the results, there is need to contextualize them (how do these compare to previous research? if there are differences, why would those differences exist?).	We have removed the lines where results were restated
3. Line 186. This sentence is too broad (i.e., "the realm of rare diseases in Tanzania"), narrow it down.	We have narrowed it down as recommended
4. Line 187, Where? in Tanzania?	Yes, we have specified that now
5. Lines 186-196. The focus is being lost from the specifics of this survey to the broader topic of rare disease. This survey is not assessing the burden of rare diseases in Tanzania. Suggest to keep the discussion focused to the results of the study.	We agree with the suggestion and have removed the lines focusing on the broader topic of rare diseases
6. Lines 197-199, rephrase for clarity.	We reviewed and felt the lines dont add further value, hence removed.
7. Line 200. This registry seems to be coming "out of the blue" - need to discuss it in intro and methods/results (as available) to give context to this sentence (add more context about the registry in the discussion as well).	We agree, hence removed it from here, have put it in recommendations only
8. Line 201. "1-3" patients is not one of the categories presented in the results. Explain this.	We appreciate that observation, it was meant to be 1 – 5, however, we have now reworded

	the entire sentence to better reflect the findings
9. Line 202. Results presented here do not correspond to results presented previously. Please correct.	We have added the percentages of the 2 categories – 11 – 20 and > 20, that gives 25%
10. Lines 205-211. Make sure that these results are the same that were presented earlier in the paper.	We have confirmed, they are the same, we have reworded for clarity
11. Lines 216-218. How would this be in Tanzania?	We have included that
12. Line 220. Delete "This was also mirrored in their responses."	Removed
13. Line 221. Specify "similar reports."	We have adjusted to findings rather than reports
14. Line 226. Specify "similar interventions."	Have changed to: capacity building options
15. Line 231. Specify "similar approaches" in all these 3 last items compare results from this study with reports from the literature, specifically, what is similar, what is different and offer potential explanations for differences.	We have added further detail
16. Line 237. Delete "this is an expected finding."	Deleted as recommended
17. Lines 253-261. Move to the appropriate sections of the discussion where those issues are being compared to HIC.	We appreciate the suggestion and have moved the section as advised
18. Line 262. Remove "few." There are more than a few limitations.	Removed as advised
19. discuss how using WhatsApp may have excluded people who don't have access to it OR do not feel comfortable using it OR are not "tech savvy" - use appropriate descriptor for the last.	This suggested is relevant and has been included in limitations
20. Line 274, start with "our findings suggest" as opposed to "it is evident."	Replaced as recommended
21. The conclusions is mostly repetitive of results. Rewrite to explain one solid conclusion and recommendation that can be derived from the results. It is not advisable to focus on a phone app as that was not the main focus of the study.	We have reviewed and rewritten the conclusions and recommendations.
Reviewer #1: The author addressed all the comments provided and I recommend the	We appreciate the reviewers feedback and support

paper to be pubished for widely dissemination as the findings are very important and have positive impact in policy implication to inform intervention in rare disease	
Reviewer #3: Despite having given the authors the opportunity for a major revision, I regret to report that:<br><br>(i) Some of the responses to my comments have been really inconsistent. (ii) This paper has serious methodological weaknesses that cannot be remedied at this stage. (iii) The contribution is, quite frankly, poor and confusing for publication in an impact journal.	We acknowledge the reviewer's sentiments. We have made every effort to respond to the reviewer's comments shared previously and we regret that we were not able to meet the reviewer's expectations.
