# Supplementary material for: Rare diseases in children—Knowledge, experiences and challenges faced by pediatricians in Tanzania
Source: PLOS Glob Public Health. 2026 May 13;6(5):e0006435. doi: 10.1371/journal.pgph.0006435 (PMC13170965; doi:10.1371/journal.pgph.0006435)
Supplement: Attachment — Submitted filename: RESPONSE TO REVIEWERS.docx [file pgph.0006435.s005.docx]

| **REVIEWER/EDITOR COMMENT** | **RESPONSE TO COMMENT** |
| --- | --- |
| EDITOR COMMENTS | |
| Thank you very much for submitting your manuscript to PLOS Global Public Health. Better understanding challenges faced by pediatricians in Tanzania in caring for children with rare diseases is an important global public health issue.  Reviewers #2 and #3 had recommendations for strengthening the manuscript, including clarifying some specific methodological details, including the complete questionnaire/survey instrument as an appendix, and expanding on the discussion section. Comparing your findings to any available data from other lower-middle income countries would be particularly valuable. | We thank the editor for sharing our concern on the importance of rare diseases in children as a global public health concern  We have addressed the comments from the reviewers and included the survey tool as supporting information. We have expanded the discussion and added further comparisons to literature |
| I would also advise elaborating a bit more on the limitations section of the discussion. Notably, given that the response rate was 52%, in what specific ways might the findings not be reflective of all pediatricians in Tanzania? For example, the majority of the respondents (79%) were under the age of 45 years. Is this representative of the population of pediatricians in Tanzania, or was the study biased toward younger respondents? If so, that might have implications for some of the findings (such as the preference for a smartphone or tablet application). | Thank you for your insightful feedback. We acknowledge the limitations posed by the response rate of 52%, which may affect the generalizability of the findings to all pediatricians in Tanzania and have addressed it further in the limitations.  We appreciate your guidance in refining this section and will ensure that these considerations are reflected in the discussion. |
| REVIEWER 1 COMMENTS | |
| A very good and important study highlighted the unmet research area on rare disease as explained by author to be the first study in the country which will inform the public policy on the rare study. It highlighted how the disease is of importance especially to healthcare workers. The validated tools adapted from Malaysia help to validate the findings for public use. It is among the importance paper to inform the policy and proper government planning | We thank the reviewer for highlighting the importance of the study and how it can inform public policy. |
| REVIEWER 2 COMMENTS | |
| What was the determinant that led to the selection of the Australian instrument for the survey? Was it culturally and contextually fit for purpose? | Thank you for your comment regarding the selection of the Australian instrument for our survey. The choice of this instrument was based on the comprehensive questions that aligned with the objectives of our research, making it a suitable foundation for adaptation.  In ensuring cultural and contextual relevance, modifications were made to tailor the survey to the Tanzanian healthcare context. We have included this information in the methodology |
| Was the survey tool pilot tested? If so, any adaptations made? | Yes the survey tool was pilot tested by administering it to paediatric residents to ensure the meaning and content of the questions was clear and modifications were made based on the input received. This information has been included in the methodology |
| How were paediatricians contacted? By phone call? email message? WhatsApp? | The questionnaire was shared as an online link circulated through the WhatsApp social media platform of the paediatric professional association – Paediatric Association of Tanzania (PAT). To encourage participation, zonal representatives were informed to disseminate information and paediatricians were also contacted directly. This information has been elaborated in the methodology section |
| Might it be possible to have a map illustrating the zones and regions where your respondents came from? | Yes Map added illustrating the zones and regions. |
| Might it be possible to have table 3 in form of a graph for ease of understanding? | yes refer to Figure 2 |
| The same recommendation is made for table 4 | yes refer to Figure 3 |
| Any data from other low-middle income countries in the same area of interest that you can compare your findings with? | Thank you for your insightful comment. This has been included in the Discussion section. |
| Please highlight recommendations for policy and future research | Thank you for your input this has been highlighted in the discussion section |
| REVIEWER 3 COMMENTS | |
| Page 2 lines 37, 38... The statement made is not in accordance with what is known as a ‘rare disease’, rather there is little clinical experience in its manifestations and it is necessary to rely especially on genetic/molecular studies to confirm the diagnosis and consequent treatment of the entity. | Thank you for this input. The statement is revised as recommended |
| Page 2 lines 45, 46... Write the bibliographic reference for this statement. | The statement has been revised, and a bibliographic reference has been added. |
| Pag 3 lines 47-50... The wording becomes somewhat monotonous due to the frequency of the verb ‘to report’ in the incorporation of incidence data. | The sentence has been revised |
| Pag 3 lines 63-65... Explain the meaning of ‘Red flags’. | Red flags are pointers for rare diseases; clarification has been included in the statement. |
| Pag 5 line 100... Item 2 ‘Education and knowledge of rare diseases’ is not specified, this item of the questionnaire needs to be better described. | This has been revised |
| Pag 5 line 103... Item 5 ‘Use and availability of resources’ is not clear, what does it refer to specifically? | This has been revised |
| Page 5 line 104...To specify item 6 ‘Perceptions and practices in the management of rare diseases’. | This has been revised |
| Pag 5 lines 106-108, The term ‘paediatric’ is written instead of ‘pediatric’, correct it or decide on the same version of writing for the whole text | This has been revised |
| Pag 5 lines 108-109, With reference to the questionnaire/survey, it would be necessary to have access to its complete visualisation as an annexed document, like the other 2 incorporated. I think it is a mistake not to present the reader with the complete questionnaire, thus also answering the questions raised above. | This will be submitted as a supplementary document. |
| Some explanation - a sentence - on the statistical handling of the data and the criteria for its application would be missing. | We thank the reviewer for noticing this omission and we have included it |
| This section of Material and Methods seems to be poorly worked out | This has been revised with more clarification |
| One of the limitations of the study is the low participation of paediatricians, only 52% of the total sample. This may affect the analysis and conclusions of the study | Thank you for your insightful feedback. We acknowledge the limitations posed by the response rate of 52%, which may affect the generalizability of the findings to all pediatricians in Tanzania.  Despite these limitations, the study provides valuable insights into the challenges faced by paediatricians regarding rare diseases  We appreciate your guidance and will ensure that these considerations are reflected in the discussion. |
| Page 6 line 121... Clarify the statement in the sentence that the 168 paediatricians who answered the questionnaire represent 52% of the study population. | This is based on the number of paediatricians who were using the WhatsApp platform at the time of data collection |
| Pag 7 line 131... How was this ‘<5% of the population in Tanzania is affected by rare diseases’ obtained, the bibliographic reference would be missing. Could it be from Zurynski Y et al 2017? In Tanzania there is still no official registry known for rare diseases. | This is the response from the participants where most of them believed that rare disease affect less than 5% of the population |
| Table 1 row 3... Sort the data correctly. Clarify the meaning of ‘paediatric specialist’, use appropriate terminology to avoid confusion. | This is noted and has been corrected to avoid confusing terminology |
| Table 2 rows 1 and 2... Participants seem to give similar answers to both questions. Treatment in equal proportions between diagnosed and undiagnosed cases is insubstantial. If it is not diagnosed as a rare disease, it is difficult to assess the comparison and the result is also meaningless. | We appreciate this insight from the reviewer. However, the aim was to describe the frequency with which paediatricians provide care for patients with diagnosed rare diseases and those in whom they have not made definitive diagnosis. The similar responses to both questions highlights that for the rare diseases that are diagnosed, an almost equal number remain undiagnosed |
| Table 2 row 3... Some disease should be specified for each system or apparatus exposed, even if only provisionally. | We appreciate this suggestion. However, since the target population for this study was paediatricians, the assumption is that they have good understanding of different systemic diseases, hence examples were not provided |
| Pag 9 lines 140-144... These data should be better incorporated in a table. | This is appreciated and we have included a chart. |
| Table 4 row 6 and 7... This option does not seem to be applicable in a context where it is difficult to confirm the diagnosis of a rare disease as in Tanzania | Even though diagnostic confirmation is difficult, clinical diagnosis is still relevant and can form the basis of referral and support groups |
| In general, the wording has some repetitions of words, and could be changed to synonyms to obtain more fluent sentences. | We appreciate this input and have adjusted wordings as much as possible |
| Pag 12 lines 189-192... These statements are not accurate - 19% and 25% are not the same - and should be justified with more objective data. | This has been adjusted as per the reviewers input |
| Pag 13 lines 193-195... It is important to soften the sentences avoiding words with absolute meaning such as ‘lack’, better to write ‘reduced’, ‘inadequate’ or ‘limited’. | This is noted and has been modified |
| Conclusions would have to be rewritten more accurately, few and set out in list form to make it easier for the reader to understand their conclusive importance. | We appreciate this input and have reorganised conclusions and recommendations into more succinct paragraphs |
| Revise the entire bibliography to correct the title of the journals with the corresponding acronyms and page spacing. | This has been adjusted to use the correct acronyms. |
| Please consider to add this reference: Mashala EI, Brunet-Llobet L, Lapitskaya A, Balsells-Mejía S, Mrina O, Miranda-Rius J. Bone disease and oromaxillofacial disorders: a cross- sectional study in a Tanzanian pediatric population. Orphanet J Rare Dis. 2025;20(1):77. Published 2025 Feb 17. doi:10.1186/s13023-025-03563-0 | We thank the reviewer for the suggestion. We have reviewed the reference and although it highlights important information on oral maxillofacial disorders in Tanzania, we do not feel that it is not an appropriate citation relevant to our study on rare diseases |
