# Supplementary material for: Rare diseases in children—Knowledge, experiences and challenges faced by pediatricians in Tanzania
Source: PLOS Glob Public Health. 2026 May 13;6(5):e0006435. doi: 10.1371/journal.pgph.0006435 (PMC13170965; doi:10.1371/journal.pgph.0006435)
Supplement: Attachment — Submitted filename: RESPONSE_TO_REVIEWERS_auresp_2.docx [file pgph.0006435.s007.docx]

**RESPONSE TO REVIEWERS**

| **COMMENT** | **RESPONSE** |
| --- | --- |
| 1. Confirm whether the data and text presented in this manuscript have not previously been published. Cover letter states that findings build upon previous published research. Clarification of this would be appreciated | We acknowledge the query and apologise for the confusing statement in the cover letter. What we meant is that our findings add to the existing knowledge on rare diseases which is predominantly from the global north. Ours now builds on this by sharing evidence from the global south. We have made edits to the cover letter to reflect this |
| 2. Confirm whether this manuscript (or any portion thereof) is being considered for publication elsewhere | We confirm that this manuscript or any portion is NOT being considered elsewhere for publication. Have indicated this in the cover letter. |
| 3. Use American English spelling. | This is noted and we have adjusted |
| 1. Rephrase first sentence for clarity. Suggested to rephrase to something like this "Diagnosing and treating rare diseases in children remain major challenges for pediatricians across the world." | This suggestion is appreciated and has been incorporated |
| 2. The second sentence state that the research gap is to understand how often Tanzanian pediatricians are met with rare diseases, yet the next sentence focuses the paper into other issues such as knowledge, challenges in treatment, etc. Which is the gap and the focus of this paper? | We thank the editor for highlighting this. We have edited to keep the focus on knowledge, practices and challenges |
| 3. Fourth sentence, suggest replacing "This" with "This study presents the results of a..." | This suggestion is accepted and the sentence has been amended |
| 4. How many total pediatricians are there in Tanzania? What proportion of those are in the WhatsApp group? | We have now included this information |
| 4.a. - for the manuscript, not the abstract, make sure to discuss selection bias - how do pediatricians get added to the WhatsApp group? Who decides, and under what criteria, who is admitted into the WhatsApp group? Is it possible that a non-pediatrician is in the WhatsApp group? How do the pediatricians in the WhatsApp group differ from pediatricians outside the group? Why use WhatsApp group and no other more commonly used participant recruitment? - Some of this is already addressed in lines 100-107 but not all, make sure that all of this is adequately addressed. | This suggestion is appreciated and we have discussed it further in the methodology |
| 5. for the manuscript, not the abstract, how do you reconcile that such a high proportion (88%) of pediatricians have encountered rare diseases in their practice, when, by definition, rare diseases show extremely low frequency in either incidence or prevalence? | We acknowledge the query raised. Although these diseases are rare individually, together they are found in 6 – 8% of the population as we mentioned in the introduction, this is in keeping with our findings, where majority of the respondents had encountered a suspected rare disease in their practice, showing that their overall incidence/prevalence is not that rare. This is now reflected in the discussion |
| 6. For the manuscript, discuss how a phone app with information will meet the need of lack of access to diagnostic tests. | The phone app was the recommended resource that paediatricians favoured – above websites and print material to bridge the knowledge gap. This has now been reflected in the recommendations. |
| 7. Discuss the limitations of the study in the abstract. | We have briefly discussed them |
| II. INTRODUCTION.  1. The citation offered for the definition of rare diseases does not link to a European definition of rare diseases. Either add a citation for the European definition OR move the citation to the right location in the sentence and add a citation for the European definition in the right location of the sentence. Also, please cite either peer-reviewed literature OR a widely accepted textbook on rare diseases for definition, as opposed to a website. See, for instance, https://doi.org/10.1016/j.jval.2015.05.008 | We appreciate the suggestion and we have included the recommended citation which provides definitions used globally |
| 2. Citation 2 (Adachi et al) contains information that is either contradictory or different or lacking to what is stated in the text (esp. regarding number of rare diseases and percentage of global population affected by it). Please update the information in the sentence OR add appropriate citation (if new citation is added, ensure that numbering is updated AND that the new citation is either more recent or more rigorous on its methodology than Adachi et al). | We acknowledge the discrepancy and have adjusted the text to reflect what is in the paper by Adachi |
| 3. Sentences in lines 37-40 are missing citation. Please add. | Have modified the sentence and added the citation |
| 4. Citation 3 in line 44 does not match the text of the sentence. Ensure that all citations match the sentence correctly. | We acknowledge the error and have changed the citation |
| 5. Line 44, specify what "our setting" mean. | We have adjusted the wording to reflect what we mean |
| 6. Rephrase line 61 for clarity. | We have rephrased this |
| 7. Line 62, comma after "Australia" is missing. | Have now included the comma |
| 8. Lines 62-66, explain how the information from these studies reflect conditions in which adequate knowledge exist OR put the information in the right context. | We have adjusted the initial line so that it no longer reflects on conditions of adequate knowledge |
| 9. Line 68, replace "red flags" for appropriate medical term. | We appreciate the suggestion, however, we have used the terminology based on how the authors of the paper described it. |
| 10. Lines 73-74, text almost verbatim from Stoller - please rewrite this paragraph. | We have rewritten as per the suggestion |
| 11. Please specify who are the members of THGO. Try to contrast membership, mandate and activities of AKRDF and THGO for the reader to understand the context of the field in Tanzania better. | We have included these |
| 12. Sentence ending in line 89 is missing citation. | We have added the citation |
| 13. As mentioned above, clarify research gap and study aim AND ensure consistency. | We have amended to reflect the aims consistently |
| 14. Line 95 - is it certain that this data WILL inform medical education? If not, then replace will by another appropriate modal verb or appropriate sentence. | This is acknowledged and we have adjusted it |
| III. MATERIALS AND METHODS  1. Add sub-headings. At minimum, Setting and Study Population, Sample Size and Sampling Methodology, Data Collection Instruments, Data Collection Process, Data Manipulation and Data Analysis, Ethical Considerations. | We have included relevant sub-headings as suggested |
| 2. Line 98, delete "of" before "conducted." | Acknowledged and deleted |
| 3. Line 99. Suggest to place "mainland and Zanzibar" in parenthesis. | Acknowledged and parenthesis added |
| 4. Line 101, add citation for the number of members in PAT. | This was informal communication with PAT leadership and has been included as such |
| 5. Line 105. Review "who are the group," it does not seem to make sense in this sentence. | The word “in” was missing and has been added |
| 6. Line 105, do you mean "about" instead of "involving"? | Yes, and we have adjusted the wording |
| 7. Line 109. Replace "in the survey done in Australia" for a more formal writing style. | We have replaced it for a more formal structure |
| 8. Line 111. Explain the "modification" process employed to adapt the survey to the Tanzanian context. | We have briefly explained these modifications |
| 8.a. what frameworks or theories guided this process? | The modification was mainly based on appropriate geographic, regulatory and resource related terminologies hence there was no specific frameworks or theories |
| 9. Line 112. Does the "questionnaire" refer to the original (i.e., Australian) OR the modified (i.e., Tanzanian) context? | It refers to the modified one, we have now specified that |
| 10. Lines 113-119. Ensure consistency of use between "participant" and "pediatrician" - stick to one or the other. | We acknowledge this and have modified to use pediatrician consistently |
| 11. Line 120. Replace "pilot tested" for "pilot-tested." | Replaced |
| 12. Line 120. Specify how many pediatricians participated in the pilot testing (and how were they selected). | We have included this |
| 12.a explain in detail the process, methodology and theoretical foundation of the pilot testing process. | We have now explained this process. There was no specific theoretical foundation we used. |
| 13. What was learned from the pilot testing? Did some question need to be further adjusted? What input was received from participants in the pilot testing? Explain here. | We have now explained this |
| 14. Describe eligibility criteria. | We have added an explanation of this |
| 14a. Were pilot testing participants allowed or not allowed to take part of the survey? Why or why not? | Pilot testing was done on senior paediatric trainees who were not eligible to participate in the survey |
| 15. Line 125, replace "to" with "and" before "4th." | Replaced as suggested |
| 16. What software was used to collect survey data? | We used Microsoft excel, this has been specified in the text |
| 17. Line 126. How was incomplete data handled? | We have now explained this |
| 18. Line 131. Clarify what is meant by "implied" informed consent. Were participants asked for consent as part of the online survey? Show a screenshot of the survey's first page. | We have further explained this and provided a screenshot as additional file. |
| 19. Describe data protection and cybersecurity steps taken to ensure privacy and confidentiality of data. | We did not take any specific measures related to cybersecurity, however data was kept confidential by avoiding use of identifiers and being accessible only to the 3 study investigators. |
| IV. RESULTS  1. Line 138. Text states "most regions" - clarify how many regions (out of how many are represented). | We have adjusted this to reflect zones rather than regions as per the figure 1 |
| 2. Figure 1 shows % from regions, how does this compare to the % of PAT members per region? | It corresponds – we have included this |
| 3. Line 139. The word "zones" is used whereas previously the word "regions" was used. Clarify and keep consistency. | We have clarified and used zones consistently |
| 4. Line 145. Describe which fields of pediatric subspecialty are represented. | We did not ask them to specify the subspecialty field, the question asked them to choose between general paediatrics or sub-speciality paediatrics with a third option of other. In some cases, people populated other to describe their relevant sub-specialisation |
| 4. Line 147 - Table 1. Add "missing" category for the "years of experience as a pediatrician" variable. | Added as advised |
| 5. Line 148 - Table 1. Use formatting where only top and bottom lines for the table and for the headings is shown - eliminate lines in the middle (between rows and between columns) and the sides. | Formatted as per advice |
| 6. Line 150. Contrast participants' beliefs (re: % of Tanzanian population affected by rare diseases) with reality. | This has now been added in the discussion |
| 7. Line 153. Word "subspecialties" is used, whereas previously the word "specialties" (or derivatives) has been used. Recommend consistent use of "sub-specialties" (and derived words) across the document. | We have adjusted this throughout the document |
| 8. Line 155. Better explained what is meant by "the commonest rare conditions." | We have adjusted the statement to say the most frequently encountered rather than commonest |
| 9. Line 158 - Table 2. Format as suggested above. | Formatted |
| 10. Line 158 - Table 2. "Number of patients ... without ... diagnosis" adds up to 99%. Please correct. | Adjusted |
| 11. Line 158 - Table 2. "Types of ..." does not add up to 168 nor to 100. Explain with a footnote. | Explained |
| 12. Line 158 - Table 2. "... most recent..." does not add to 100%. Please correct. | Corrected |
| 13. Line 160. Specify 97% of what? | Specified |
| 14. Line 167. How many participants have attended CME or specialty/sub-specialty conferences where they could have been exposed to appropriate rare diseases content? Was this explored? | WE have included the percentage that had attended conferences/training |
| 15. Line 168. Specify, half of what? | Have specified |
| 16. Line 170 and in general. Do not start sentences with numbers nor acronyms. | Have adjusted |
| 17. Line 171. There is a reference to "domains" but domains explored have not been previously introduced. Explain domains (and theories the work draws from) in the methods section. | The wording has been changed to sections instead of domains – reflecting on how the questionnaire was organised |
| 18. Figure 3 cannot be understood as is. Add appropriate contextual notes, legends, better categories, etc. to understand what information is being presented. | We have modified the figure to improve presentability |
| 19. Line 174. Does this refer to currently available resources (i.e., apps or online websites) or resources they wished they had but they do not have access to right now? | It refers to those they were most likely to use if available (whether or not they are currently available). We have reworded the text to reflect this |
| 20. Figure 3 does not match context described in text. | We have revised figure 3 to match the context |
| 21. Figure 4 does not match context described in text. | Have replaced figure 4 with the correct figure |
| 22. Need to better explain and connect survey to results. Need to add phrases like, "when asked about XXXX" participants showed "XXX" | We have rephrased the results as per the recommendation |
| 22a. in the methods section describe the structure of the survey. For instance, Part 1 asked questions about XXXX such as "XXXXXX;" Part 2 asked XXXXXX, etc. | We appreciate the suggestion, and have explained in the methods |
| V. DISCUSSION.  1. Lines 180-181. Before the suggestion was made to clarify research gap and survey intent. Make sure that this line remains consistent with any changes made. | We have changed to maintain consistency |
| 2. Lines 181-185. There is no need to restate the results, there is need to contextualize them (how do these compare to previous research? if there are differences, why would those differences exist?). | We have removed the lines where results were restated |
| 3. Line 186. This sentence is too broad (i.e., "the realm of rare diseases in Tanzania"), narrow it down. | We have narrowed it down as recommended |
| 4. Line 187, Where? in Tanzania? | Yes, we have specified that now |
| 5. Lines 186-196. The focus is being lost from the specifics of this survey to the broader topic of rare disease. This survey is not assessing the burden of rare diseases in Tanzania. Suggest to keep the discussion focused to the results of the study. | We agree with the suggestion and have removed the lines focusing on the broader topic of rare diseases |
| 6. Lines 197-199, rephrase for clarity. | We reviewed and felt the lines dont add further value, hence removed. |
| 7. Line 200. This registry seems to be coming "out of the blue" - need to discuss it in intro and methods/results (as available) to give context to this sentence (add more context about the registry in the discussion as well). | We agree, hence removed it from here, have put it in recommendations only |
| 8. Line 201. "1-3" patients is not one of the categories presented in the results. Explain this. | We appreciate that observation, it was meant to be 1 – 5, however, we have now reworded the entire sentence to better reflect the findings |
| 9. Line 202. Results presented here do not correspond to results presented previously. Please correct. | We have added the percentages of the 2 categories – 11 – 20 and > 20, that gives 25% |
| 10. Lines 205-211. Make sure that these results are the same that were presented earlier in the paper. | We have confirmed, they are the same, we have reworded for clarity |
| 11. Lines 216-218. How would this be in Tanzania? | We have included that |
| 12. Line 220. Delete "This was also mirrored in their responses." | Removed |
| 13. Line 221. Specify "similar reports." | We have adjusted to findings rather than reports |
| 14. Line 226. Specify "similar interventions." | Have changed to: capacity building options |
| 15. Line 231. Specify "similar approaches" in all these 3 last items compare results from this study with reports from the literature, specifically, what is similar, what is different and offer potential explanations for differences. | We have added further detail |
| 16. Line 237. Delete "this is an expected finding." | Deleted as recommended |
| 17. Lines 253-261. Move to the appropriate sections of the discussion where those issues are being compared to HIC. | We appreciate the suggestion and have moved the section as advised |
| 18. Line 262. Remove "few." There are more than a few limitations. | Removed as advised |
| 19. discuss how using WhatsApp may have excluded people who don't have access to it OR do not feel comfortable using it OR are not "tech savvy" - use appropriate descriptor for the last. | This suggested is relevant and has been included in limitations |
| 20. Line 274, start with "our findings suggest" as opposed to "it is evident." | Replaced as recommended |
| 21. The conclusions is mostly repetitive of results. Rewrite to explain one solid conclusion and recommendation that can be derived from the results. It is not advisable to focus on a phone app as that was not the main focus of the study. | We have reviewed and rewritten the conclusions and recommendations. |
| Reviewer #1: The author addressed all the comments provided and I recommend the paper to be pubished for widely dissemination as the findings are very important and have positive impact in policy implication to inform intervention in rare disease | We appreciate the reviewers feedback and support |
| Reviewer #3: Despite having given the authors the opportunity for a major revision, I regret to report that:  (i) Some of the responses to my comments have been really inconsistent. (ii) This paper has serious methodological weaknesses that cannot be remedied at this stage. (iii) The contribution is, quite frankly, poor and confusing for publication in an impact journal. | We acknowledge the reviewer’s sentiments. We have made every effort to respond to the reviewer’s comments shared previously and we regret that we were not able to meet the reviewer’s expectations. |
