# Supplementary material for: Rare diseases in children—Knowledge, experiences and challenges faced by pediatricians in Tanzania
Source: PLOS Glob Public Health. 2026 May 13;6(5):e0006435. doi: 10.1371/journal.pgph.0006435 (PMC13170965; doi:10.1371/journal.pgph.0006435)
Supplement: Attachment — Submitted filename: response_to_reviewers_auresp_3.docx [file pgph.0006435.s008.docx]

| **COMMENT** | **RESPONSE** |
| --- | --- |
| Reviewer #1:   The Manuscript is getting in shape for publication as the author addressed all the comments given by reviewers | We appreciate the reviewers endorsement and thank them for helping us improve on the write up |
| Reviewer #4: | |
| To my knowledge, this is the first national-level study from Tanzania examining rare diseases from the perspective of pediatricians, and it addresses a substantial evidence gap. The manuscript is ethically sound, transparently reported, and written in clear English. The dataset is openly available and complies with PLOS data-sharing requirements. | We thank the reviewer for the positive remarks on the importance of the manuscript and the quality of the work |
| However, while the study is publishable in principle, it currently falls short of the analytical rigor expected for a Research Article in PLOS Global Public Health. The work is largely descriptive, and the available data are underutilized. This does not warrant rejection, but it does require major revision. | We acknowledge the reviewers sentiment and every effort has been made to improve the rigor as per the feedback from the reviewers.  The study was designed as a descriptive one with the main aim being to get an overall understanding of the burden of rare diseases in Tanzania from the perspective of paedatricians |
| Reviewer #5: | |
| The manuscript tackles an important, under-studied topic, but clarity, rigor, and interpretability are limited. It offers valuable insights but needs substantial revision to strengthen structure, methodological transparency, data interpretation, and alignment of conclusions with findings. | We thank the reviewer for highlighting the importance of the topic. We have further revised the manuscript based on the detailed feedback received (Details in the rows that follow and in the revised manuscript submission). We hope this improves the work substantially |
| Abstract: Unstructured; PLOS requires labeled sections (Background, Methods, Results, Conclusions). | I have reviewed the author guidelines and it does NOT require the specific labelled sections. It says: **While the Abstract is conceptually divided into three sections (Background, Methodology/Principal Findings, and Conclusions/Significance), do not apply these distinct headings to the Abstract within the article file.**  We have reviewed and improved the flow of the abstract to reflect these sections |
| Background lacks clear justification for Tanzania and a defined knowledge gap. | We appreciate the importance of highlighting the knowledge gap in the abstract. We have explicitly indicated this in the abstract as follows:  **Gap: The knowledge, experiences and challenges faced by pediatricians in Tanzania is not known.**  However due to word count limitations, we could not explain the justification in detail in the abstract but have elaborated in the main text |
| The Methods section in the abstract is missing. Key details such as study design, population, sampling approach, survey instrument, data collection period, and type of analysis are not described. | We have modified the abstract to ensure the methods clearly stand out as follows: **This study used a nationwide cross-sectional online survey to describe the knowledge of pediatricians in Tanzania on rare diseases, their experiences, and the challenges they face in treating these children.**  The design is specified as cross- sectional  The population is indicated as paediatricians in Tanzania  Its mentioned as a nationwide survey hence no sampling  The instrument is mentioned as an online survey  The type of analysis is mentioned as descriptive  Due to word limits, we could not specify data collection period, but its detailed in the main body |
| Results include interpretive statements, and conclusions are broad with advocacy-style language. Minor grammatical errors and repetition reduce clarity. Minor grammatical and typographical errors (e.g., “60% of felt unprepared” knowledge, experiences and challenges … is not known”). | We appreciate the keenness of the reviewer who has identified the typographical errors. We have reviewed and corrected these.  We have reviewed the results statements to remove the interpretive tone  The conclusions and recommendations are derived from the study findings and we have revised to make them more specific |
| Introduction: Overly long with global context not fully linked to study aims. Rare diseases are not clearly defined, and the Tanzanian healthcare context is underexplored. The research gap is implied rather than explicit, and rationale for a survey approach is not justified. The introduction would benefit from greater conciseness, clearer definition of key concepts, stronger contextualization to Tanzania, and a more focused articulation of the study’s knowledge gap and rationale | We acknowledge the reviewers observations and suggestions.  We have made the introduction more concise, reduced the length, and provided explicit gaps as well as rationale  Rare disease definitions have clearly been given with an explanation that these definitions vary globally |
| Methods: The Methods section provides a general overview of the study design and data collection process; however, several methodological details are missing or insufficiently developed, limiting reproducibility and assessment of rigor. Important methodological details related to study design, sampling, and eligibility criteria are inadequately described. Use of the PAT WhatsApp group may introduce selection bias. Questionnaire validation and pilot testing are limited and not fully described. Inclusion and exclusion criteria are not fully specified. Data collection process: Measures taken to prevent duplicate responses are not described. No data management plan (e.g., data security, access control) is described. Data analysis is restricted to descriptive statistics without subgroup analysis, and Excel use is not justified. | We acknowledge the gaps highlighted. Some areas are already captured including the study design which is mentioned as cross-sectional. The sampling has not been discussed since this was a nationwide survey and the eligibility is specified as: **pediatricians working in Tanzania (Mainland and Zanzibar) who are members of Paediatric Association of Tanzania (PAT) and were included in the official PAT WhatsApp social media platform**  We agree that the use of the WhatsApp group may introduce bias and we have included that as a possible limitation. However, we have also discussed that 90% of paediatricians are part of the group.  Details on the questionaires development and pilot testing are described in detail. We have moved this section on data collection process to the section on data collection instrument for further clarity  We have now included how we avoided duplicate responses.  Details on data security are described under the ethics section.  Since the aim of the study was descriptive, the data analysis methods were also descriptive. The study was not powered for any sub-group analysis hence this was not done.  We acknowledge that Excel is not a standard data analysis software. However, due to the basic data analytic methods and the use of descriptive charts and graphs, the study team decided that excel would be adequate without need for more advanced software. |
| Results: Entirely descriptive and self-reported; multiple-response questions and denominators are unclear. Subgroup analyses are missing, and potential biases (selection, recall) are not addressed. Figures and tables require clearer captions and consistent reporting. | We acknowledge the descriptive nature of the results and the self reporting; however, this was the aim of the study – to describe the experiences of the paediatricians hence it has to be self-reported.  We did not do sub group analysis since that was not the study aim and the sample size was not powered for this type of analysis  We have reviewed figures and tables to ensure captions are clear and the reporting is consistent |
| Discussion: Overly descriptive and repetitive. Critical interpretation is limited, causal statements from cross-sectional data are inappropriate, and the Tanzanian healthcare context is underexplored. Biases and methodological limitations are incompletely discussed. Capacity-building recommendations are not critically evaluated for feasibility. Pilot testing was conducted among senior pediatric residents rather than practicing pediatricians, which may limit its relevance; this limitation is not acknowledged. | We appreciate the input on the discussion.  We have critically reviewed the discussion and are confident that it explores the Tanzanian healthcare context and does not infer causality. We have clearly highlighted limitations including the methodological ones  Regarding recommendations, we accept that we have not evaluated feasibility; however that is beyond the context of this paper.  Pilot testing was done using senior residents and the aim of pilot testing was to ensure feasibility and appropriateness to the Tanzanian context, we believe that it was appropriate without becoming a limitation. |
| Conclusion/Recommendations: Summarizes key findings but repeats Results/Discussion. Self-reported findings should be interpreted cautiously. Recommendations need prioritization, clearer stakeholder roles, and discussion of feasibility. Study limitations should be explicitly acknowledged, and patient/family perspectives could be better linked to clinical care implications. | We appreciate the suggestions. We have included stakeholder roles in the recommendations and added on the patient/family perspectives to improve clinical care  The study limitations are now explicitly reworded. |
| Reviewer #6: | |
| Generally, the manuscript is well-written; however, the limitations do not address the small pre-testing sample, which may have overlooked questionnaire flaws and contributed to validity concerns | We appreciate the reviewers suggestion and have included the small pretesting sample as a limitation. |
| Reviews from PDF document attached | |
| The introduction would benefit from a clearer conceptual or analytical framework (e.g., health workforce readiness, diagnostic cascade, or systems-level bottlenecks).  Currently, the background reads as a narrative review rather than a problem-driven framing leading to specific analytical questions.  While the aim is stated, it remains broad (“knowledge, experiences and challenges”).•  The manuscript would benefit from explicit research questions or domains | We thank the reviewer for the recommendations. We have reviewed the introduction to make it more analytic and highlight the existing challenges related to rare diseases globally then focusing on the situation in Tanzania.  We acknowledge the importance of a conceptual framework in research; however, for this study, we did not utilise a specific framework since the study was an exploratory one.  We agree with the reviewer that the aim is broad; however those were the 3 domains we were exploring: knowledge, experiences and challenges |
| While ~90% coverage of pediatricians is claimed via the WhatsApp group, this is based on **informal communication**, not verifiable registry data.  The manuscript should more explicitly acknowledge **selection bias**, especially digital access bias.  A 52% response rate is acceptable for online surveys but **not interrogated analytically**.  No comparison between respondents and non-respondents is attempted.  Analysis is limited to **descriptive statistics using Excel**.  No stratified analysis (e.g., by years of experience, sub-specialty, sector, or zone) is performed, despite sufficient sample size. | We agree with the reviewers valid concerns about informal communication. This was provided by PAT leadership who checked with the national medical council  We recognise the importance of selection bias in our study and have clearly highlighted it as a limitation  We acknowledge the lack of comparison, however since this was an anonymised voluntary survey, we could not derive such comparisons  We agree with the reviewer that the analysis is descriptive; however this was the overall objective of the study. We are cognizant that excel is not a standard data analytic software but it was most appropriate since we focused on descriptive analysis and the use of charts and tables.  We did not do a stratified analysis since the sample size was not powered for these. Additionally the data points in different categories were few and this would limit the interpretation of results of any sub group analysis |
| “Rare disease” is based on **self-perception**, not standardized case definitions.  This should be explicitly framed as *perceived or presumed rare diseases* throughout the manuscript.  The study is methodologically adequate but analytically underpowered. | We accept the reviewer’s recommendation and have included presumed rare disease as appropriate across the whole manuscript |
| The results stop at frequencies and percentages.  No attempt is made to identify **patterns or gradients** (e.g., generalists vs subspecialists, junior vs senior clinicians).  The frequency of encountering rare diseases may reflect **diagnostic uncertainty**, not confirmed diagnoses.  This distinction is not sufficiently emphasized in the results section.  The result section is clear and readable but missed deeper insights. | We acknowledge that the data is descriptive; however, the aim of this study was to get an overall perspective hence we did not do subgroup comparisons.  WE acknowledge this and have included it in the discussion |
| The discussion reiterates findings rather than **interrogating mechanisms** (e.g., why training gaps persist, structural constraints in diagnostics).  Recommendations (registries, curricula, networks) are sensible but **not prioritized, costed, or linked to feasibility** within Tanzania’s health system.  The role of government, insurers, or regional collaborations could be elaborated.  While generally cautious, some language implies causality (e.g., training gaps → poor outcomes) that cannot be established from cross-sectional self-report data. | We appreciate the reviewers feedback and have improved upon the discussion.  We are grateful for this suggestion and have improved upon the recommendation by specifying stakeholders and prioritizing actions.  We acknowledge the implied causality and have reviewed the text to ensure it is not inferred. |
