# Supplementary material for: Rare diseases in children—Knowledge, experiences and challenges faced by pediatricians in Tanzania
Source: PLOS Glob Public Health. 2026 May 13;6(5):e0006435. doi: 10.1371/journal.pgph.0006435 (PMC13170965; doi:10.1371/journal.pgph.0006435)
Supplement: Attachment — Submitted filename: response_to_reviewers_auresp_4.docx [file pgph.0006435.s009.docx]

| **COMMENT** | **RESPONSE** |
| --- | --- |
| Lines 16-19: Slightly awkward phrasing; could be “…WhatsApp group, where most pediatricians are registered. | We appreciate this suggestion and have made the changes |
| Line 91-92: Please provide a reference to support the statement about AKRDF’s role in Tanzania’s rare disease movement. | The reference has been added as requested |
| Lines 102-106: The sentence is long and not clear; consider splitting it. | It has been split as per the reviewer’s recommendation |
| The introduction is overly long (five pages) and would benefit from substantial condensation. The authors should streamline the section, remove redundancies, and focus on the most relevant background to improve clarity and readability. Make the introduction a 2-page | We acknowledge this concern, and have further reduced the background section. It is now just over 2 pages without track changes |
| Figures 2, 3, and 4 do not have titles. Please provide appropriate titles and captions for clarity." | The titles of the figures are listed in the manuscript text |
| Figure 1 does not appear essential to the manuscript and could be removed to improve focus and conciseness." | We acknowledge the reviewer’s suggestion, however we included the map based on a previous reviewer’s suggestion that it would be useful to see the various areas where the paediatricians came from and we feel it adds value to readers who may not be familiar with Tanzania’s Geography |
