# Supplementary material for: Rare diseases in children—Knowledge, experiences and challenges faced by pediatricians in Tanzania
Source: PLOS Glob Public Health. 2026 May 13;6(5):e0006435. doi: 10.1371/journal.pgph.0006435 (PMC13170965; doi:10.1371/journal.pgph.0006435)
Supplement: Attachment — Submitted filename: response_to_reviewers_auresp_5.docx [file pgph.0006435.s010.docx]

| **Reviewer comment** | **Response to reviewers** |
| --- | --- |
| I also couldn't make visible links between the AKRDF group mentioned early on the introduction (lines 77) to how the study was then conducted. Please explain how the groups reference relates to the study | We thank the reviewer for the relevant concern. The AKRDF was mentioned to give a background on the status of the rare disease movement in Tanzania. AKRDF was also the funder of the study (which is mentioned in funding source) |
| Please format figure 4 to provide better contrast in reading. | We have reviewed the figure 4 and it was formatted using the NAAS tool as recommended by the journal |
| The authors should still review minor grammar and style issues, such as hyphenation in ‘low- and middle-income countries’ and sentence structure, to ensure consistency and readability. | This has now been revised as per reviewer recommendation |
